# Intrinsic Inflammatory Signaling in Life and Death of Podocytes

**DOI:** 10.3390/ijms27219923

**Published:** 2026-11-05

**Authors:** Danila E. Bobkov, Sofya A. Konopleva, Dmitry A. Osin, Oleg N. Demidov, Veronika S. Mihailovskaya

**Affiliations:** 1 Department of Immunobiology and Biomedicine, Sirius University of Science and Technology, 354340 Sirius, Russia; s.konopleva.s@mail.ru (S.A.K.); d-osin@inbox.ru (D.A.O.); demidov.on@mail.ru (O.N.D.); 2 Institute of Cytology, Russian Academy of Sciences, 194064 St. Petersburg, Russia; 3 INSERM UMR1231, University of Burgundy, 21078 Dijon, France; 4 Institute of Ecology and Genetics of Microorganisms, Ural Branch of the Russian Academy of Sciences, 614081 Perm, Russia

**Keywords:** podocyte, glomerular filtration barrier, podocyte-intrinsic inflammation, NF-κB, cGAS–STING, NLRP3 inflammasome, Rho GTPases, regulated cell death, podocyte senescence, proteinuria

## Abstract

Podocytes are terminally differentiated components of the glomerular filtration barrier whose structural integrity depends on coordinated signaling between the slit diaphragm, actin cytoskeleton, mitochondria, and stress-response machinery. Evidence from cultured podocytes, experimental kidney disease, human tissue, and selected podocyte-specific genetic models indicates that podocytes can execute intrinsic inflammatory responses and, in defined settings, contribute to local inflammatory amplification. This review examines how NF-κB, p38 MAPK, JAK–STAT, cGAS–STING, NLRP3 inflammasome, and Rho-family GTPase signaling interact with cytoskeletal remodeling, mitochondrial dysfunction, senescence, regulated cell death, and podocyte detachment. Particular attention is given to the distinction between podocyte-specific evidence and findings obtained from whole-kidney analyses, global genetic models, or pharmacological interventions. The available evidence supports a context-dependent network in which inflammatory signaling may support adaptation but, when sustained or dysregulated, can contribute to barrier dysfunction and irreversible podocyte loss. We also assess therapeutic strategies targeting inflammatory sensors, kinases, inflammasome components, cytoskeletal regulators, and metabolic stress pathways. Most remain preclinical, emphasizing the need for podocyte-targeted delivery, rigorous cell-specific validation, and human translational studies.

## 1. Introduction

### 1.1. Overview of Podocyte Biology and Function

**Figure 1:**
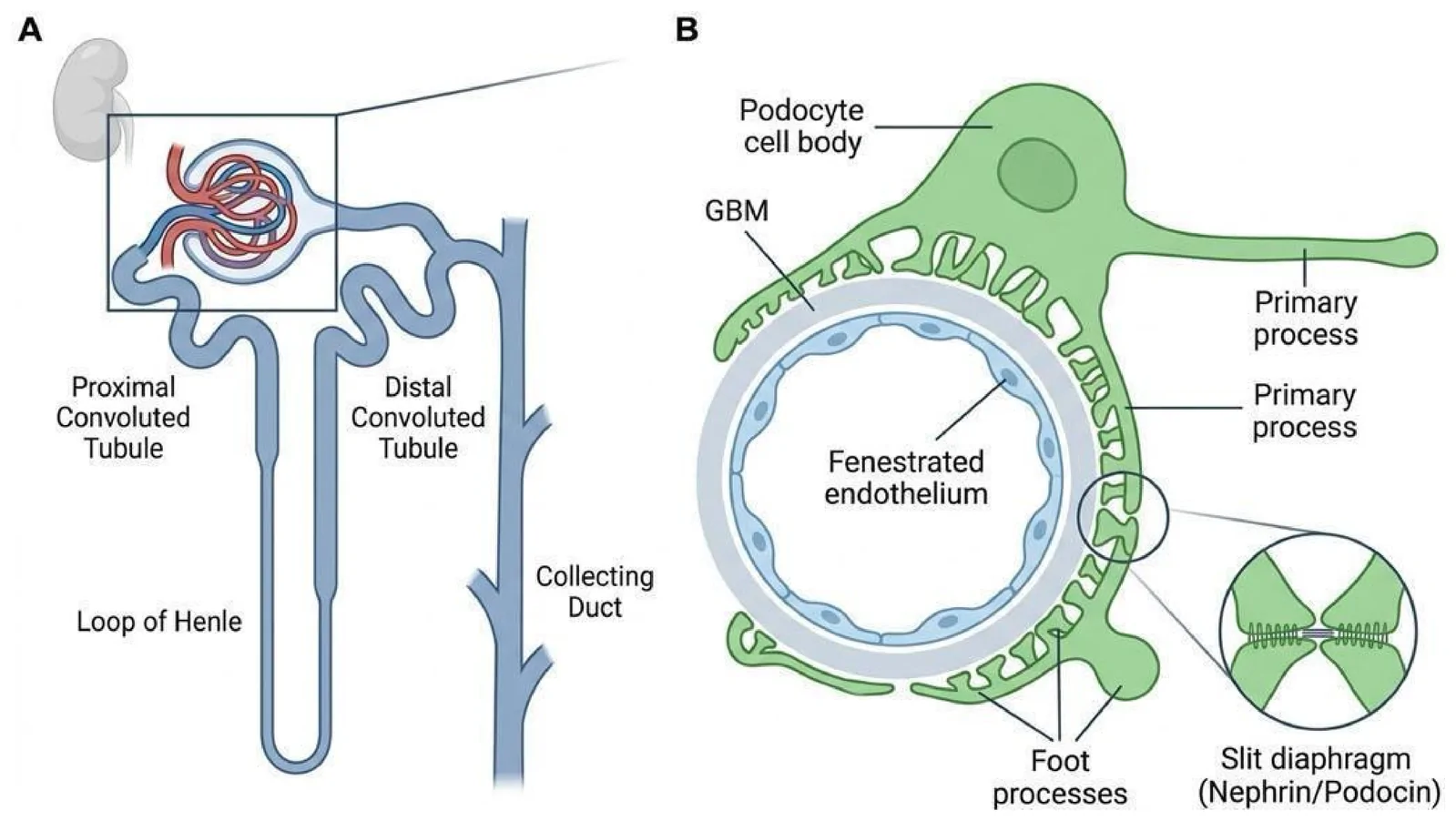
Anatomy of the podocyte within the glomerular filtration barrier. (**A**) Schematic representation of the nephron with the glomerulus highlighted, indicating the position of podocytes at the outer aspect of the glomerular capillary tuft. (**B**) Magnified view of a single glomerular capillary loop in cross-section, showing fenestrated endothelial cells lining the capillary lumen, the glomerular basement membrane (GBM), and a podocyte with its cell body, primary processes, and highly branched foot processes. Foot processes interdigitate and are connected by slit diaphragms containing nephrin and associated transmembrane proteins, supported by membrane-associated scaffolding proteins such as podocin. Together with the GBM and fenestrated endothelium, this specialized junctional system contributes to glomerular filtration selectivity.

Podocytes are highly specialized, terminally differentiated epithelial cells of the visceral layer of Bowman’s capsule. They form the outermost cellular component of the glomerular filtration barrier and are indispensable for the long-term maintenance of glomerular permselectivity and renal homeostasis (Figure 1) [1,2]. Their unique architecture comprises a cell body, primary processes, and numerous interdigitating foot processes, which are connected by slit diaphragms. The slit diaphragm is a specialized signaling and cell-junction complex containing nephrin, podocin, CD2AP, NEPH1, and ZO-1, which functionally links the filtration barrier to the actin cytoskeleton and intracellular signaling networks [3,4].

Mature podocytes have a highly limited capacity for cell division and replacement. Consequently, podocyte loss is a critical and frequently irreversible event in the progression of chronic glomerular disease. Experimental podocyte-depletion models demonstrate a direct quantitative relationship between the extent of podocyte loss, proteinuria, focal segmental glomerulosclerosis (FSGS), and declining renal function [5,6,7].

Historically, podocytes were regarded primarily as passive structural elements of the glomerular filter. Over the past decades, this concept has been substantially revised. Contemporary evidence indicates that podocytes are metabolically active and signal-responsive cells capable of sensing a broad spectrum of mechanical, metabolic, and immune stimuli. Podocytes express innate immune receptors, including Toll-like receptors (TLRs), and can respond to pathogen- and damage-associated molecular patterns (PAMPs and DAMPs) [8]. In diverse experimental models of glomerular injury, activation of inflammatory and stress-responsive pathways within podocytes has been implicated in altered slit-diaphragm protein expression, actin cytoskeletal remodeling, loss of adhesion to the glomerular basement membrane (GBM), and regulated cell-death responses [9,10,11,12]. In addition, microRNA-mediated control of injury signaling is increasingly recognized [13].

Inflammatory signaling in podocytes rarely occurs in isolation. Instead, podocyte injury, local cytokine and chemokine production, complement activation, and immune-cell recruitment can participate in self-sustaining pathological loops that perpetuate glomerular inflammation and structural injury [14]. In diabetic kidney disease and experimental immune glomerular injury, these processes can contribute to progressive podocyte dysfunction and loss, thereby compromising the integrity of the glomerular filtration barrier [9,10,15]. Despite intensive investigation of inflammation in renal pathology, the mechanisms by which inflammatory signaling pathways directly drive podocyte death remain an active area of research. Increasing attention has focused on regulated cell-death modalities, including apoptosis, pyroptosis, and necroptosis, and on their contribution to podocyte injury. Understanding the molecular links between inflammatory signaling and podocyte death is essential for developing therapeutic strategies aimed at preserving podocyte number and limiting disease progression.

### 1.2. Defining Podocyte-Intrinsic Inflammation

Accumulating evidence indicates that podocytes possess immune-like sensing and signaling capabilities, enabling them to detect danger-associated stimuli and mount intrinsic inflammatory responses [8,9]. In this review, podocyte-intrinsic inflammatory signaling denotes inflammatory sensing, signal integration, and effector responses operating within podocytes, irrespective of whether the initiating stimulus originates inside or outside the cell. Podocyte-autonomous describes a response supported experimentally as executable without ongoing input from another cell type. Podocyte-initiated inflammation refers to a tissue-level process in which podocyte-derived mediators or damage signals contribute to responses in neighboring or recruited cells. These properties overlap but are not interchangeable: an extracellular cytokine can elicit a podocyte-intrinsic response, whereas podocyte-specific genetic protection does not, by itself, establish independence from intercellular signaling.

**Figure 2:**
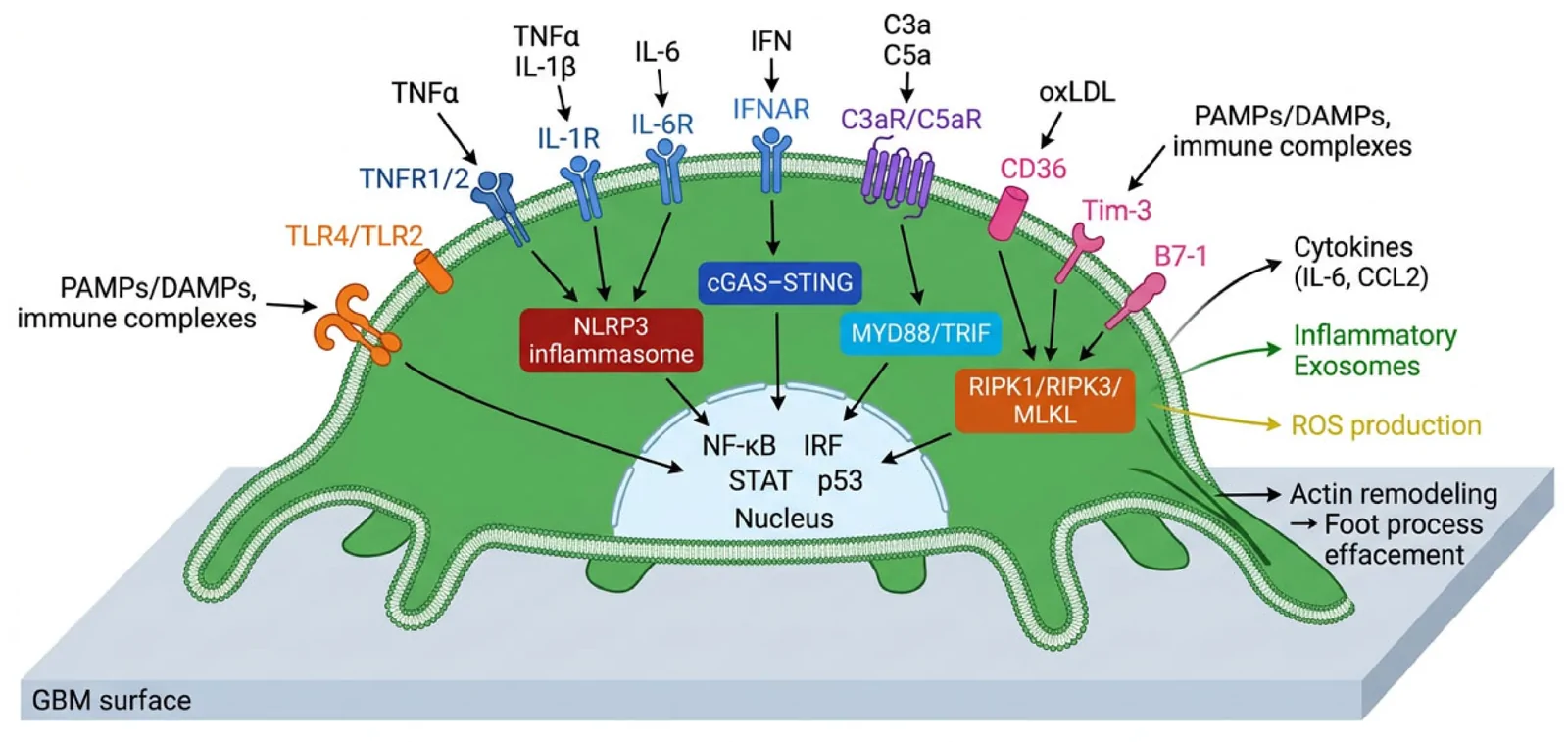
The immune-like podocyte: membrane receptors, intrinsic innate immune signaling, and effector outputs. A podocyte positioned on the outer surface of the GBM is exposed to circulating, glomerular, and local danger-associated signals. The schematic summarizes membrane receptors and immune-associated molecules reported in podocytes across different experimental and disease settings. These include pattern-recognition receptors such as TLR2 and TLR4; cytokine receptors such as TNFR1, TNFR2, IL-1R, IL-6R, and IFNAR; complement receptors such as C3aR and C5aR; and additional molecules such as CD36, Tim-3, and B7-1/CD80. Their expression and functional relevance are context dependent, and podocyte B7-1/CD80 expression remains controversial. Extracellular inflammatory, metabolic, complement-derived, and danger-associated cues can activate distinct receptor-dependent pathways or induce intracellular stress. Receptor-derived and intracellular stress signals converge on innate immune signaling platforms, including the NLRP3 inflammasome, cGAS–STING, MyD88/TRIF-associated pathways, MAVS, and RIPK1/RIPK3/MLKL signaling. These pathways regulate NF-κB-, IRF-, and STAT-dependent transcriptional responses and can promote cytokine and chemokine production, release of extracellular vesicles and reactive oxygen species (ROS), actin-cytoskeletal remodeling, foot-process effacement, and disruption of the glomerular filtration barrier. The diagram integrates mechanisms reported across different models and does not imply that all components are simultaneously active or equally established in every disease context.

Operationally, podocyte-intrinsic inflammatory signaling can be triggered by extracellular cues or intracellular stress arising during hyperglycemia, dyslipidemia, immune-complex-associated injury, complement activation, mechanical stress, or toxin exposure. These stimuli act through podocyte receptors, ion channels, and intracellular sensing and signaling systems, including cytokine and complement receptors, pattern-recognition receptors, the cGAS–STING pathway, and NLRP3-associated signaling. These inputs converge on transcriptional and post-transcriptional networks comprising NF-κB, p38 and other MAPKs, JAK–STAT, interferon-regulated factors, and epigenetic and epitranscriptomic regulators, which together reprogram podocyte behavior [9,11,13]. It is important to note that the activation of these signaling pathways can be either protective or pathogenic, depending on disease stage, intensity and duration of the stimulus. As summarized in Figure 2, podocytes integrate extracellular inflammatory, metabolic, complement-derived, and danger-associated cues through membrane receptors and cytosolic sensors, thereby coupling innate immune signaling to cytoskeletal remodeling, inflammatory output, and filtration-barrier dysfunction.

Conceptually, this framework comprises three related processes. First, podocytes sense extracellular inflammatory cues and intracellular disturbances through their own receptors and stress-response machinery. Second, signal integration links inflammatory pathways to podocyte structural and functional systems, including the slit diaphragm, actin cytoskeleton, mitochondria, autophagy, and regulated cell-death machinery. Third, in defined settings, podocyte-derived cytokines, chemokines, extracellular vesicles, and senescence-associated secretory phenotype (SASP) factors may influence neighboring or recruited cells. This intercellular propagation is a potential consequence of podocyte-intrinsic signaling rather than a requirement for its definition.

To evaluate this propagation phase, the podocyte-derived signal, its release, the recipient cell, and the resulting functional response should be considered separately. Changes in mesangial expansion, immune infiltration, or tubular injury after podocyte manipulation do not, by themselves, identify the mediator responsible or establish direct intercellular signaling. Likewise, exposure to a recombinant ligand demonstrates recipient-cell responsiveness but does not establish physiological delivery from podocytes.

Within this framework, podocyte-intrinsic inflammation emerges as an important determinant of podocyte cellular fate, modulating the balance between adaptive stress responses and maladaptive injury across proteinuric diseases, including diabetic kidney disease, obesity-related glomerulopathy, FSGS, lupus nephritis, minimal change disease, and autoimmune glomerular disorders. Transient and appropriately resolved signaling may support short-term adaptation to injury, whereas sustained or dysregulated inflammatory activation can promote senescence, apoptosis, pyroptosis, and other regulated cell-death programs, ultimately contributing to irreversible podocyte loss [13,16,17,18].

Podocyte-restricted genetic perturbations support a functional contribution of the targeted pathway within the podocyte compartment in selected models. Complementary isolated-cell and intercellular-transfer experiments can help determine whether a response is autonomous or depends on signals from other cell types [9,15,19]. The following sections examine NF-κB, p38 MAPK, JAK–STAT, Rho-family GTPase, cGAS–STING, and NLRP3-associated signaling as interconnected components of this intrinsic inflammatory network.

### 1.3. Literature Search and Evidence Appraisal

This narrative review was informed by literature searches in PubMed, Scopus, and Web of Science, last updated on 28 October 2026. The cited literature spans 1997–2026. Searches combined “podocyte” or “glomerular epithelial cell” with pathway-related terms, including “NF-κB,” “p38 MAPK,” “MK2,” “JAK–STAT,” “Rho GTPase,” “Rac1,” “RhoA,” “Cdc42,” “ROCK,” “cGAS–STING,” and “NLRP3,” and outcome-related terms, including “inflammation,” “oxidative stress,” “senescence,” “apoptosis,” “pyroptosis,” “necroptosis,” “ferroptosis,” “PANoptosis,” and “detachment.” Disease-focused searches included diabetic kidney disease, focal segmental glomerulosclerosis, lupus nephritis, and membranous nephropathy.

Studies were selected for their relevance to inflammatory signaling within podocytes, its structural and cell-fate consequences, or therapeutic modulation. Primary mechanistic studies were prioritized, while reviews and clinical guidelines provided broader biological and clinical context. Studies in other cell types were included when necessary to explain conserved mechanisms, with their relevance to podocytes explicitly qualified. Publications without a substantive connection to these questions were excluded.

Evidence was interpreted according to the experimental model, cell specificity, and functional readouts. Evidence from human tissues and clinical studies, cultured human or animal podocytes, kidney organoids, podocyte-specific animal models, global genetic manipulations, and systemic pharmacological interventions was distinguished wherever relevant. Conflicting findings were compared with regard to species, differentiation state, culture conditions, stimulus intensity and duration, genetic strategy, and assays used. Greater weight was given to convergent functional evidence and cell-specific perturbations than to marker abundance alone; unresolved discrepancies were retained explicitly. The review provides a critical narrative synthesis rather than a systematic review or meta-analysis.

Interpretation also depends on the experimental model. Species, cell source, immortalization, differentiation state, culture conditions, and stimulus concentration can influence podocyte phenotype and inflammatory responses. Findings in cultured cells therefore require validation in complementary systems before being generalized to mature human podocytes in vivo. Conditional genetic manipulation is not necessarily adult-inducible: developmental effects, chronic adaptation, and the timing of recombination must be considered. Human relevance and causal specificity are separate dimensions of evidence, and neither whole-kidney pathway activation nor improvement in albuminuria identifies the responsible cellular compartment by itself.

## 2. Inflammatory Signaling Pathways in Podocytes

### 2.1. NF-κB and p38 MAPK Signaling in Podocytes

NF-κB and p38 MAPK function as complementary arms of the podocyte stress response. NF-κB acts primarily as a transcriptional hub, converting inflammatory and metabolic inputs into sustained changes in gene expression. p38 operates in parallel as a stress-responsive kinase, linking cellular stress to rapid cytoskeletal remodeling, inflammatory mediator production, and cell death. Despite their distinct molecular mechanisms, these pathways are co-activated by overlapping stimuli and create coordinated responses that determine podocyte fate.

At the level of the glomerular filtration barrier, NF-κB can be activated in all major glomerular cell populations, including podocytes, mesangial cells, and endothelial cells. However, podocytes, as highly specialized epithelial cells, display a distinctive combination of NF-κB-dependent pro-inflammatory programs and survival pathways. As a result, NF-κB in podocytes exerts a classical dual role: moderate and transient activation may support cellular adaptation and survival, whereas sustained hyperactivation drives podocytopathy, proteinuria, and progression of glomerular disease [19,20,21,22,23,24].

Similarly, p38 MAPK exhibits context-dependent functions in podocytes. While often associated with stress-induced injury and apoptosis, p38 can also restrain pathological pathways and preserve podocyte-endothelial interactions in certain settings. The sections below describe the upstream activators, downstream effectors, and in vivo evidence for both pathways, emphasizing their integration and shared role in driving podocyte dysfunction when chronically activated.

#### 2.1.1. Complementary Functions and Upstream Activation of NF-κB and p38 MAPK in Podocytes

Podocytes express multiple receptor systems that can engage NF-κB signaling, including Toll-like receptors, cytokine receptors such as TNFR1/2 and IL-1R, RAGE, angiotensin II type 1 receptors, and complement receptors (C3aR and C5aR). The expression and functional relevance of individual TLR family members vary across species, disease contexts, and experimental models; therefore, a uniform podocyte TLR repertoire should not be assumed [9,20,22,25,26,27,28]. Loss of nephrin itself activates NF-κB, illustrating how a primary structural defect of the slit diaphragm can be converted into a sustained inflammatory transcriptional program in podocytes [26,29]. This establishes a mechanistic link between slit-diaphragm disruption, inflammatory transcriptional reprogramming, cytoskeletal remodeling, and progressive podocyte injury.

In experimental LPS-induced podocyte injury, activation of the TLR4/MyD88/NF-κB and calcineurin/NFAT pathways increases angiopoietin-like protein 4 (ANGPTL4) expression. ANGPTL4, in turn, contributes to cytoskeletal remodeling, synaptopodin loss, and proteinuria; pharmacological or siRNA-mediated inhibition of either pathway attenuates ANGPTL4 induction and podocyte injury [30]. During septic acute kidney injury, this NF-κB hyperactivation drives podocyte apoptosis and inflammatory injury. The pathogenic role of NF-κB in this setting is underscored by the protective effect of miR-129-5p, which limits NF-κB activation by targeting the HMGB1/TLR2-TLR4 axis, thereby reducing podocyte apoptosis and improving survival [31].

In cultured mouse podocytes, angiotensin II reduced Akt and p65 NF-κB phosphorylation, decreased cell viability, and increased caspase-9 expression and apoptosis. Pharmacological activation of PI3K partially restored Akt/p65 phosphorylation and attenuated apoptosis, whereas PI3K inhibition aggravated the injurious response, consistent with a protective role of the PI3K/Akt/NF-κB survival axis in this experimental setting [27]. Consistent with these in vitro observations, chronic angiotensin II infusion in rats was associated with reduced nephrin expression and increased podocyte apoptosis in vivo [32]. In a distinct model, angiotensin II-induced podocyte apoptosis was linked to AT1R-dependent endoplasmic reticulum stress and activation of the PKC-δ/p38 MAPK pathway, highlighting that angiotensin II can engage multiple context-dependent injury programs in podocytes [33].

Complement-derived anaphylatoxins can act directly on podocytes. In primary membranous nephropathy, C3a/C3aR signaling has been implicated in podocyte injury, synaptopodin loss, cytoskeletal abnormalities, and impairment of filtration-barrier function. Although C5aR signaling and downstream PI3K/Akt, MAPK, and NF-κB pathways may contribute in selected disease settings, their podocyte-intrinsic roles should be described as context dependent rather than as a single established complement–TLR4 convergence pathway [28,34].

In diabetic nephropathy, hyperglycemia sustains canonical NF-κB signaling in podocytes through several convergent, partly self-amplifying mechanisms. Increased REDD1 expression promotes GSK3β activation, NEMO phosphorylation, and IKK complex assembly, leading to IκBα degradation, NF-κB nuclear translocation, and induction of CCL2, IL-1β, and other pro-inflammatory mediators [35]. In parallel, hyperglycemia increases podocyte expression of receptor activator of NF-κB (RANK), which promotes a p65-dependent oxidative and inflammatory program. This pathogenic role of RANK should be interpreted in a diabetic context, as RANK–RANKL signaling has also been implicated in adaptive pro-survival responses in other models of podocyte injury. In diabetic podocytes, p65 binds to the promoter regions of Nox4 (NADPH oxidase 4) and p22phox (phagocyte-type NADPH oxidase cytochrome b light chain), enhancing NADPH oxidase expression, ROS generation, and the production of inflammatory mediators [17]. High glucose-induced ROS can in turn reinforce canonical NF-κB activation, thereby establishing a feed-forward circuit that links metabolic stress to oxidative injury, cytokine expression, and podocyte dysfunction. In parallel, cytokine- and interferon-responsive JAK–STAT pathways may converge with NF-κB-dependent transcriptional programs, further broadening the inflammatory response in diabetic podocytopathies.

NF-κB can also be activated from within podocytes, independent of surface receptors. Intracellular Ca^2+^ imbalance activates calpain, a protease that directly degrades IκBα, releasing NF-κB for nuclear translocation. In cyclin G-associated kinase-deficient podocytes, enhanced calpain activity causes excessive IκBα degradation, driving NF-κB hyperactivation, podocytopathy, and glomerulosclerosis. Calpain inhibition or podocyte-specific deletion normalizes NF-κB activation and markedly reduces proteinuria and sclerosis [36]. Thus, podocyte NF-κB integrates both receptor-driven signals and intracellular stress, with calpain serving as a key modulator in toxic and metabolic injury.

In podocytes, IKK-dependent NF-κB activation can occur in parallel with stress-activated kinases such as p38, JNK, and ERK in response to selected inflammatory and metabolic stimuli [9,22,35], creating a coordinated transcriptional and post-transcriptional response to TNF, LPS, hyperglycemia, and complement activation. Rather than acting as a universal parallel output of every stimulus, p38 MAPK appears to function as a stress-responsive kinase that couples selected injurious cues to cytoskeletal remodeling, inflammatory gene expression, and apoptotic signaling.

In experimental models of diabetic podocyte injury, high extracellular glucose promotes NADPH oxidase- and mitochondria-derived ROS generation in podocytes, resulting in p38 MAPK and caspase-3 activation within a pro-apoptotic signaling program and contributing to early podocyte depletion in diabetic mouse models [10]. Albumin exposure activates p38–MK2 signaling in podocytes and induces COX-2 expression, whereas pharmacological inhibition of p38 or MK2 attenuates injury-associated cytoskeletal changes in experimental podocyte models [37,38]. Intracellular albumin overload also induces ER stress and activates a PKC-δ–p38–caspase-12 pathway, promoting podocyte apoptosis; this response is reduced by p38 inhibition [39].

TGF-β1 can activate MAPK pathways in cultured podocytes and promote loss of structural proteins and inflammatory responses. Inhibition of p38 attenuates injury-associated changes in selected experimental settings [40]. TGF-β/Smad7-dependent apoptosis provides an additional mechanism of podocyte injury [41]. Angiotensin II activates an AT1R-dependent ER stress/PKC-δ/p38 MAPK cascade in podocytes, leading to increased Bax/Bcl-2 ratio, NHE1 activation, and apoptosis; both AT1R blockade and p38 inhibition are protective in this experimental setting [33].

Hyperglycemia also activates p38 MAPK in podocytes. Activated p38 phosphorylates the nephrin C-terminus, facilitating PKCα recruitment and β-arrestin2-dependent nephrin endocytosis; pharmacological p38 inhibition preserves nephrin surface expression and attenuates albuminuria in experimental hyperglycemia [42]. In adriamycin (ADR)-induced podocyte injury, loss of plectin activates an integrin α6β4/FAK/p38 MAPK cascade. FAK-dependent p38 activation is associated with podocyte apoptosis and F-actin cytoskeletal disruption, whereas restoration of plectin expression attenuates pathway activation and protects against podocyte injury [43].

By contrast, direct evidence that TLR4/LPS or IL-1β induces p38-dependent podocyte injury remains less extensive than the evidence for TLR–NF-κB signaling. These pathways should therefore be described as plausible contributors to p38 activation rather than as fully established podocyte-intrinsic p38 mechanisms. Together, NF-κB and p38 MAPK function as interconnected components of the podocyte stress response. NF-κB predominantly coordinates inflammatory and pro-survival transcriptional programs, whereas p38 MAPK prominently links cellular stress to cytoskeletal remodeling, apoptotic signaling, and the induction of selected inflammatory mediators. Their concurrent activation by metabolic, hemodynamic, or immune stimuli can integrate transcriptional and structural injury responses; when sustained or insufficiently resolved, this signaling convergence may promote podocyte dysfunction, detachment, and progressive loss.

#### 2.1.2. NF-κB-Dependent Inflammatory Programming in Podocytes

Building on the receptor- and stress-dependent inputs outlined above, canonical NF-κB activation in podocytes converges on the IKK complex, composed of IKKα, IKKβ, and the regulatory subunit NEMO/IKKγ. Although individual stimuli use distinct proximal adaptors and ubiquitin-dependent signaling modules, IKKβ-mediated phosphorylation of IκBα promotes its ubiquitin-dependent degradation and the nuclear translocation of p65/p50 dimers [44,45,46]. In canonical NF-κB signaling, IKKβ phosphorylates IκBα, promoting its ubiquitin-dependent degradation and release of p65/p50 dimers. Nuclear p65/p50 complexes then induce context-dependent transcriptional programs involved in inflammation, cell survival, matrix remodeling, and stress adaptation [44,45,46].

The non-canonical NF-κB pathway is activated by a restricted subset of TNF receptor superfamily members, including RANK, BAFF-R, and CD40. Unlike canonical signaling, it does not rely on MyD88 or TRADD. Instead, receptor activation promotes degradation of the TRAF2–TRAF3–cIAP1/2 complex that constitutively targets NF-κB-inducing kinase (NIK) for proteasomal turnover, thereby allowing NIK stabilization [21,23,47,48,49]. Stabilized NIK activates IKKα, which phosphorylates p100 and promotes its processing to p52, enabling the formation and nuclear accumulation of RelB/p52 dimers [47,48,49]. These dimers regulate a distinct transcriptional program linked to chronic inflammation, fibrosis, and long-term adaptation to persistent stress [21,23]. Although non-canonical NF-κB signaling is well established in immune and stromal cells, its cell-autonomous functions in podocytes remain incompletely defined.

NF-κB activity in podocytes is further modulated by intracellular kinases such as GSK3β. GSK3β influences p65 phosphorylation and target-gene selection, thereby shifting NF-κB-dependent responses from pro-survival programs, including *Bcl-xL*, towards pro-inflammatory programs, including *Ccl2* and *Cxcl1* [22]. In diabetic nephropathy, hyperglycemia-induced REDD1 promotes GSK3β-dependent canonical NF-κB activation in podocytes, contributing to sustained inflammatory mediator expression and podocyte injury. Independently, GSK3β hyperactivity can impair Nrf2-dependent antioxidant responses and promote oxidative stress and premature podocyte senescence in experimental type 2 diabetic kidney disease [35,50]. Genetic or pharmacological inhibition of GSK3β attenuates injury-associated NF-κB signaling and improves podocyte outcomes in experimental settings; however, pathway-selective and podocyte-targeted therapeutic strategies remain to be established [35,50].

NF-κB signaling is subject to regulation by microRNAs. In immune cells, miR-146a functions as a canonical negative-feedback regulator of innate inflammatory signaling by targeting IRAK1 and TRAF6, two key components of the TLR/IL-1R–NF-κB pathway [51]. Podocyte studies likewise support a protective role for miR-146a in glomerular injury. Global miR-146a deficiency accelerates streptozotocin (STZ)-induced diabetic glomerulopathy and albuminuria, in association with increased expression of ErbB4 and Notch1 [13]. Podocyte-specific deletion of miR-146a increases susceptibility to low-dose LPS, nephrotoxic serum nephritis, and STZ-induced diabetic kidney disease, resulting in enhanced proteinuria and glomerular injury. These effects are associated with increased ErbB4/EGFR signaling and enhanced TGF-β/Smad3 activity [52]. miR-129-5p has also been reported to limit LPS-associated podocyte injury by attenuating the HMGB1/TLR2/TLR4/NF-κB axis [31]. Conversely, NF-κB can regulate the expression of selected microRNAs and long non-coding RNAs, thereby creating feedback circuits that may sustain chronic inflammatory signaling and promote podocyte injury in glomerular disease [18].

In podocytes, the NF-κB transcriptional response is context dependent. Under basal or transient stress conditions, NF-κB can support adaptive and pro-survival programs, whereas persistent injury shifts target-gene expression towards chemokines, cytokines, matrix-remodeling mediators, and regulators of cytoskeletal dysfunction [53,54]. Furthermore, the role of NF-κB depends on the stage and type of disease: it has been shown that NF-κB modulates acute inflammatory kidney injury but does not affect chronic fibrotic injury [55].

Finally, NF-κB and Notch signaling may converge in injured podocytes, where persistent activation of inflammatory and developmental programs can promote dedifferentiation, apoptosis, and filtration-barrier dysfunction [56,57]. In diabetic settings, glucose-derived advanced glycation end products can activate Notch1 signaling in podocytes and contribute to proteinuria, supporting a role for Notch activation in metabolic podocyte injury [58]. However, the molecular hierarchy and direct causal links between NF-κB and Notch activation remain unresolved across distinct podocytopathies.

#### 2.1.3. p38 MAPK as a Stress and Cytoskeletal Effector

While NF-κB functions as a major transcriptional integrator of podocyte-intrinsic inflammation, p38 MAPK acts as a stress-responsive kinase that links mechanical, metabolic, and protein-overload signals to cytoskeletal regulation, inflammatory gene expression, and context-dependent cell-death responses. But similarly to NF-κB, the role of p38 MAPK in podocytes is context-dependent.

In podocytes, activated p38α phosphorylates MK2 (MAPKAPK2), which in turn phosphorylates the small heat-shock protein HSPB1/HSP25, thereby modulating actin filament dynamics. Acute high-glucose exposure activates the p38–HSP25 pathway and supports actin cytoskeletal stability; pharmacological p38 inhibition reduces HSP25 phosphorylation, promotes F-to-G-actin conversion, and disrupts the cytoskeleton in this setting [59].

In contrast, sustained protein-overload injury can engage a maladaptive p38/MK2 program. Albumin exposure activates p38 and MK2 in podocytes and induces COX-2 expression, whereas pharmacological inhibition of p38 or MK2 reduces albumin-induced COX-2 expression and attenuates PAN-associated cytoskeletal injury [37,38]. Thus, the p38–MK2–HSPB1 axis can serve adaptive functions during acute stress but may contribute to inflammatory and structural injury when activation is persistent or combined with nephrotic stressors. This dual nature underscores that therapeutic p38 MAPK inhibition must be carefully titrated.

p38 MAPK also contributes to slit-diaphragm dysfunction in diabetic settings. Hyperglycemia-induced p38 activation promotes nephrin phosphorylation and β-arrestin2-dependent nephrin endocytosis, thereby reducing surface nephrin availability and compromising filtration-barrier integrity [42]. The p38 pathway intersects functionally with Rho GTPase networks; in diabetic podocytes, Rac1/PAK1 activation lies upstream of p38 and contributes to β-catenin-associated slit-diaphragm disruption, foot-process effacement, and proteinuria [60].

p38 MAPK also participates in several stress-induced apoptotic programs in podocytes. Intracellular albumin overload induces ER stress, as reflected by increased GRP78 and IRE1α activation, and engages a PKC-δ–p38–caspase-12 cascade that promotes podocyte apoptosis; pharmacological p38 inhibition attenuates this response [39]. Angiotensin II activates an AT1R-dependent ER stress/PKC-δ/p38 MAPK pathway, accompanied by NHE1 activation, an increased Bax/Bcl-2 ratio, and apoptosis, whereas AT1R blockade or p38 inhibition is protective in experimental podocyte models [33]. TGF-β1-induced podocyte apoptosis likewise requires p38 MAPK activation and is associated with increased Bax expression and caspase-3 activity [41]. In ADR-exposed podocytes, ROS generation precedes ASK1–p38 activation, and suppression of this pathway reduces oxidative podocyte injury [61]. Collectively, these findings identify p38 MAPK as a context-dependent convergence point through which ER stress, oxidative stress, and profibrotic signaling can promote apoptotic podocyte injury.

#### 2.1.4. NF-κB and p38 MAPK in Glomerular Disease: Evidence from In Vivo Genetic Models

Experimental and clinical studies implicate aberrant NF-κB activation in a broad range of proteinuric and inflammatory glomerular diseases, including diabetic kidney disease, FSGS, membranous nephropathy, IgA nephropathy, and immune-mediated glomerulonephritides. However, the relevant cell types, upstream triggers, and functional consequences differ substantially among disease settings [9,16,20,21]. The contribution of NF-κB to disease progression operates at two levels: systemic and local inflammation, involving immune cell infiltration and cytokine production in the interstitium and mesangium, and podocyte-intrinsic signaling that can alter the structure and function of the filtration barrier. The available genetic evidence is summarized in Table 1, which distinguishes global from podocyte-specific manipulations and highlights the context-dependent functions of NF-κB, p38 MAPK, and their associated regulatory modules in experimental glomerular disease.

Podocyte-specific genetic models have been essential for defining the contributions of NF-κB signaling within podocytes. In nephrotoxic nephritis, podocyte-specific NEMO/IKKγ-deficient mice display immune-cell infiltration comparable to that of control animals but show accelerated recovery from proteinuria and reduced glomerular expression of NF-κB-dependent pro-inflammatory chemokines. These findings indicate that podocyte NF-κB can sustain glomerular inflammatory signaling and delay functional recovery independently of changes in leukocyte recruitment [9]. Similarly, in ADR-nephropathy, podocyte-specific inhibition of NF-κB does not impair baseline kidney function but markedly attenuates albuminuria and loss of slit diaphragm proteins such as nephrin and synaptopodin after injury [19]. In a model of nephrotoxic nephritis, podocyte-specific deletion of *Myd88* has little effect on the inflammatory transcriptome and disease severity, indicating that podocyte inflammatory responses in this setting are largely independent of canonical MyD88-dependent TLR signaling [62].

In diabetic nephropathy, NF-κB in podocytes integrates signals from RANK, angiotensin II, NLRP3 inflammasome, and NADPH oxidases, amplifying oxidative stress and production of TNF, IL-1β, MCP-1, and other inflammatory mediators [16,17,18,21,27]. Podocyte-specific RANK knockout in HFD/STZ, db/db, and related diabetic models reduces albuminuria, mesangial expansion, and podocyte loss, accompanied by decreased p65-dependent transcription of NOX4 and p22phox and reduced oxidative stress, thereby confirming the importance of RANK-driven NF-κB activation in diabetic podocytopathy [17]. Podocyte-specific REDD1 deletion similarly protects against diabetic injury, as reflected by reduced albuminuria, glomerular hypertrophy, and mesangial matrix deposition, together with preservation of podocyte number and foot process architecture, consistent with REDD1–GSK3β–NF-κB as a major pathogenic axis [35].

In membranous nephropathy and passive Heymann nephritis, local immune deposits and complement activation induce NF-κB-dependent inflammatory responses in podocytes, including TRAF6/NF-κB, TLR/NF-κB, and NLRP3-linked pathways, driving proteinuria and structural remodeling of the filtration barrier [16,28,63,64]. These observations place NF-κB within a broader innate immune network that also involves complement, cGAS–STING, and inflammasome signaling. Astragaloside IV, which has potent anti-inflammatory effects, reduces proteinuria, and improves histology in passive Heymann nephritis and diabetic nephropathy while suppressing NF-κB signaling and lowering TNF, IL-6, and IL-1β expression in podocytes [16,63,64].

The role of podocyte NF-κB is not uniformly pathogenic in every form of renal injury. In renal ischemia–reperfusion injury, podocyte-specific NF-κB blockade has little effect on the severity of acute injury, immune cell infiltration, or functional recovery, whereas systemic NF-κB inhibition reduces inflammation [19,65]. These findings suggest that in purely ischemic acute kidney injury, NF-κB-dependent inflammation is driven predominantly by tubular cells and interstitial macrophages, with a limited contribution from podocytes. Thus, the pathogenic significance of podocyte NF-κB is highly disease- and context-dependent: it is central in metabolic and immune-mediated glomerulopathies but less critical in purely ischemic settings.

Other in vivo models highlight the consequences of primary structural or signaling defects that secondarily hyperactivate NF-κB in podocytes. Nephrin-deficient mice, modeling congenital nephrotic syndrome, demonstrate that nephrin loss triggers NF-κB activation [26,29]. Podocyte-specific cyclin G-associated kinase (GAK) knockout causes calpain-mediated IκBα degradation and progressive podocytopathy; additional Capns1 deletion dampens NF-κB activation and significantly improves proteinuria, survival, and glomerular morphology, highlighting the calpain–IκB–NF-κB axis as a driver of podocyte injury [36]. Global and podocyte-specific miR-146a deletion models further illustrate the importance of endogenous post-transcriptional restraint in podocyte injury. Global miR-146a deficiency accelerates STZ-induced diabetic glomerulopathy through dysregulation of the ErbB4–Notch1 axis, whereas podocyte-specific miR-146a deletion increases susceptibility to LPS-, nephrotoxic serum-, and diabetes-induced glomerular injury, with enhanced ErbB4/EGFR and TGF-β-associated signaling [13,52].

Taken together, these in vivo data show that podocyte NF-κB is a dual regulator: it is required for stress-adaptive and survival programs, yet when excessively or improperly activated by diabetes, immune complex nephritis, or structural slit diaphragm defects, it becomes a major driver of proteinuria and progressive podocytopathy [9,16,19,21]. This supports a therapeutic strategy aimed not at indiscriminate global inhibition of NF-κB, but at targeting podocyte-specific fine-tuning modules such as NEMO, REDD1, RANK, miR-146a, and the calpain–IκB axis, thereby selectively attenuating pathological inflammatory signaling while preserving basal podocyte homeostasis.

Like NF-κB, p38 MAPK functions as a context-dependent regulator of podocyte injury. Phospho-p38 is consistently elevated in podocytes and other glomerular cells across proliferative and non-proliferative glomerulonephritides, correlating with morphological damage and renal dysfunction, and is detected in human diabetic nephropathy across mesangial, endothelial, podocyte, and tubulointerstitial compartments. In experimental PAN and ADR-nephropathy, glomerular p38 activation is transient and precedes overt proteinuria, indicating an early pathogenic role. A complete germline knockout of p38α/MAPK14 is poorly suited to analysis of adult renal injury, because Mapk14−/− mice die embryonically due to severe placental defects [66,67]. Consequently, the contribution of p38α in the kidney, and specifically in podocytes, has been studied primarily in conditional tissue-specific models or via downstream components.

The most informative podocyte-specific model to date is the study by Sugioka et al. (2023), which compared pod-p38 cKO, pod-GC-A cKO, and pod-p38/GC-A double cKO mice [68]. Two-month-old pod-p38 cKO and double cKO animals had no striking baseline phenotype, but under high salt and aldosterone load the most severe injury developed in pod-double cKO mice: serum creatinine and albuminuria increased, macrophage infiltration intensified, foot process structure and nephrin/podocin expression were disrupted, and intracapillary fibrin thrombi formed, indicating secondary endothelial injury [68]. This thrombotic phenotype was driven by elevated PAI-1 (plasminogen activator inhibitor-1, which blocks fibrinolysis and promotes fibrosis). PAI-1 neutralization reduced thrombosis and mitigated damage. Surprisingly, p38α was not purely pro-injurious in this setting: it appeared to restrain PAI-1/TGF-β activation and preserve podocyte-endothelial interactions [68]. Thus, p38α can have protective functions depending on the disease context.

For downstream branches, MK2/MAPKAPK2 and MK3/MAPKAPK3 are the most studied. Systemic MK2 knockout in mice reduces TNF production by ≈90% and confers resistance to LPS-induced endotoxin shock, underscoring the central role of the p38–MK2 axis in inflammatory responses [67]. In an acute proliferative glomerulonephritis model, MK2-, MK3-, and MK2/MK3-deficient mice were compared: MK2 loss alone had modest effects, MK3 deficiency slightly worsened disease, whereas double MK2/MK3 knockout made animals more susceptible to glomerulonephritis [69]. These findings highlight that downstream p38 targets in the kidney act in a non-linear fashion, with disease outcomes depending on kinase combinations and cellular context. Podocyte-specific data for MK2/3 remain less definitive than for the Mapk14/Npr1 double knockout model; protective effects of MK2 and p38 inhibition were shown in podocyte culture rather than in vivo: the MK2 inhibitor C23 and p38 inhibitor SB203580 reduced PAN-induced injury, preserved the actin cytoskeleton, and blocked albumin-induced COX-2 upregulation [37].

These findings favor context-dependent modulation rather than uniform suppression of podocyte NF-κB and p38 signaling. Protective and pathogenic effects must be interpreted according to disease model, downstream branch, and intervention timing. Protection by a systemic inhibitor does not, by itself, identify a podocyte-restricted mechanism.

**Table 1:** Genetic models defining context-dependent NF-κB and p38 MAPK functions in podocytes, glomerular disease, and renal inflammatory injury.

Pathway/Mediator	Genetic Manipulation	Injury Model or Context	Principal Renal and Podocyte Phenotype	Reference
**NF-κB Pathway and Associated Regulators**				
NEMO/IKKγ	Podocyte-specific deletion (Nphs2-Cre)	Nephrotoxic nephritis	Renal leukocyte infiltration was unchanged; proteinuria and podocyte MCP-1 secretion were reduced, supporting a pathogenic contribution of podocyte NEMO-dependent signaling in this model	[9]
REDD1	Podocyte-specific deletion (Nphs2-Cre)	STZ-induced diabetes	Reduced albuminuria, podocyte loss, glomerular hypertrophy, and mesangial matrix expansion; attenuated REDD1–GSK3β–NF-κB signaling	[35]
miR-146a	Global deletion	STZ-induced diabetes	Increased susceptibility to diabetic kidney injury, with aggravated albuminuria, podocyte injury, and glomerular damage	[13,52]
miR-146a	Podocyte-specific deletion (Nphs2-Cre)	Low-dose LPS, nephrotoxic serum nephritis, and STZ-induced diabetes	Increased proteinuria and glomerular injury; enhanced NF-κB-dependent and ErbB4/Notch-associated signaling	[52]
NF-κB	Podocyte-specific expression of non-degradable IκBα (IκBΔN)	ADR nephropathy	Reduced proteinuria; preserved nephrin and synaptopodin expression; improved foot-process ultrastructure	[19]
NF-κB	Podocyte-specific expression of non-degradable IκBα (IκBΔN)	Renal ischemia– reperfusion injury	No substantial improvement in acute kidney injury or renal functional indices, indicating limited pathogenic relevance of podocyte NF-κB in this setting	[65]
MyD88	Podocyte-specific deletion	Nephrotoxic nephritis	Normal baseline renal morphology and function; transiently increased proteinuria after injury, indicating that MyD88-dependent signaling may also support adaptive podocyte responses	[62]
RANK	Podocyte-specific deletion	STZ-induced diabetes	Reduced albuminuria, mesangial expansion, GBM thickening, NOX4/p22phox expression, oxidative stress, and nuclear p65 accumulation	[17]
Nephrin (Nphs1)	Germline deficiency	Spontaneous congenital nephropathy	NF-κB activation in podocytes, severe proteinuria, progressive glomerulosclerosis, and impaired recovery after injury	[26,29]
Calpain-1/2 (Capns1)	Podocyte-specific deletion on a Gak-deficient background	Progressive Gak-deficient podocytopathy	Reduced calpain-1/2 activity, IκBα proteolysis, p65-dependent Gadd45β expression, proteinuria, glomerulosclerosis, and mortality	[36]
CDK5	Podocyte-specific deletion	Nephrotoxic nephritis	No developmental phenotype at baseline; increased susceptibility to nephrotoxic nephritis, podocyte apoptosis, and glomerular injury	[70]
**p38 MAPK pathway and downstream regulators**				
DUSP4	Global deletion	Diabetic kidney disease	Increased albuminuria, mesangial expansion, glomerular fibrosis, foot-process effacement, and podocyte loss, associated with sustained p38/JNK activation and increased NOX4 expression	[71]
MAPK14/p38α	Global deletion	No injury induction	Mid-gestational embryonic lethality is caused predominantly by defective placental development; adult renal phenotyping is not feasible	[72,73]
MAPK14/p38α	Podocyte-specific deletion (nephrin-Cre)	Baseline and aldosterone/high-salt challenge	No major baseline phenotype; p38α loss exacerbated injury in the context of podocyte GC-A deficiency	[68]
NPR1/GC-A	Podocyte-specific deletion (nephrin-Cre)	Aldosterone/high-salt challenge	Podocyte injury and increased susceptibility to glomerular damage	[68,74]
MAPK14/p38α and NPR1/GC-A	Podocyte-specific double deletion (nephrin-Cre)	Aldosterone/high-salt challenge	Severe albuminuria, increased serum creatinine, macrophage recruitment, foot-process injury, reduced nephrin/podocin, intracapillary fibrin thrombi, and increased PAI-1 expression	[68]
MAPKAPK2/MK2	Global deletion	LPS-induced endotoxic shock	Markedly reduced TNF production and resistance to endotoxic shock	[75]
MAPKAPK2/MK2 and MAPKAPK3/MK3	Global single and double deletion	Acute proliferative glomerulonephritis	MK2 deficiency had limited effects; MK3 deficiency modestly aggravated disease; combined MK2/MK3 deficiency increased susceptibility to glomerulonephritis	[69]

Note: Podocyte-restricted genetic manipulation supports a contribution of the targeted pathway within podocytes but does not, by itself, establish an adult-specific function or independence from intercellular signaling. Global genetic manipulation does not establish podocyte specificity; developmental timing and inducibility should be considered when interpreting each model.

### 2.2. Cytokine Signaling: JAK–STAT

The JAK–STAT signaling pathway conveys signals from a broad range of extracellular ligands, including cytokines, growth factors, and hormones, and acts as a key mediator of pathological crosstalk between immune cells and podocytes [76]. Whereas NF-κB and p38 MAPK are prominent integrators of innate immune, stress, and metabolic inputs, the JAK–STAT axis primarily translates cytokine, interferon, and selected growth-factor signals into transcriptional programs that reprogram podocyte phenotype [76]. In diabetic and immune-mediated podocytopathies, the JAK2/STAT3 axis has emerged as particularly relevant, with IL-6-driven autocrine loops amplifying and sustaining an inflammatory state [77,78,79].

#### 2.2.1. Activation of JAK–STAT Signaling in Podocytes

The JAK–STAT pathway is a classical cytokine-activated cascade. It is evolutionarily conserved and comprises the receptor-associated tyrosine kinases JAK1, JAK2, JAK3, and TYK2 and the transcription factors STAT1, STAT2, STAT3, STAT4, STAT5A, STAT5B, and STAT6. Ligand binding promotes receptor-associated JAK activation, receptor and STAT phosphorylation, STAT dimerization, nuclear translocation, and context-dependent gene transcription [76]. JAK1 and JAK2 are broadly relevant to cytokine and growth-factor signaling in intrinsic renal cells, whereas JAK3 is expressed predominantly in hematopoietic cells and transduces signals from common γ-chain cytokine receptors. TYK2 contributes to type I interferon and selected cytokine receptor pathways [76].

The prevailing cytokine milieu that shapes JAK–STAT activation in the glomerulus varies markedly between disease entities. In diabetic kidney disease, a pro-inflammatory milieu enriched in IL-6, TNF, and CCL2/MCP-1 contributes to glomerular injury. IL-6 and TNF can be produced by infiltrating monocytes/macrophages and resident kidney cells, including mesangial cells, tubular epithelial cells, and podocytes, whereas CCL2 promotes further monocyte recruitment and chronic inflammation [76,77]. In this context, glomerular JAK–STAT activation can involve IL-6-dependent JAK1/JAK2–STAT3 signaling, whereas TNF reinforces the inflammatory podocyte phenotype, in part through TNFR2-dependent MCP-1/CCL2 induction [78,80,81].

In FSGS, the pattern is more heterogeneous. A subset of patients shows activation of intrinsic TNF signaling, and transcriptomic analyses reveal JAK–STAT pathway activation in kidney tissue and peripheral blood cells [82]. Additionally, recurrent FSGS has been linked to the IL-6-family cytokine CLCF1, which increases glomerular permeability via JAK2/STAT3 [83]. In crescentic glomerulonephritis and anti-GBM nephritis, IL-6/EGFR-associated STAT3 activation dominates: IL-6 is produced by infiltrating lymphocytes and podocytes themselves, while EGF/TGF-like signals via EGFR sustain and prolong STAT3 activation in podocytes, promoting crescent formation [79,84]. Finally, in FSGS there is also a protective branch of JAK–STAT signaling: IL-15 can activate STAT5 in podocytes, and podocyte-specific Stat5 deletion worsens FSGS, indicating coexistence of pathogenic and adaptive JAK–STAT modules within the same cell [85]. This dichotomy—pathogenic STAT3 signaling versus protective STAT5 signaling—underscores the dual role of JAK–STAT signaling pathways, which is dependent on the stimulating cytokine, the type of activated STAT protein, and the stage of glomerulonephritis.

Importantly, under pathologic conditions podocytes not only respond to cytokines but also become sources of inflammatory mediators. Upon TNF stimulation, cultured podocytes sharply increase MCP-1/CCL2 secretion, predominantly via TNFR2 [80]. IL-6 stimulation activates STAT3 and upregulates STAT3 targets in podocytes (ICAM1, CCL2, IL6, and SOCS3), indicating an autocrine amplifying loop [79]. TLR4 activation by LPS or lipid A induces a broad chemokine repertoire, including CCL2, CCL7, CXCL1, and CXCL5, and to a lesser extent CCL3, CCL5, CXCL7, CXCL9, CXCL11, and CXCL13 [8]. Together, these data show that injured podocytes generate their own inflammatory secretome, in which CCL2/MCP-1, IL-6, ICAM-1-associated STAT3 targets, and TLR4-induced chemokines are particularly well documented [8,79,80,81,86].

Growth factors of the EGF family, especially EGF and HB-EGF, further amplify podocyte injury by engaging EGFR and activating JAK–STAT3 signaling. This damage is mediated less by direct cytotoxicity than by the induction of a pro-inflammatory and proliferative podocyte phenotype: EGFR/STAT3 activation drives STAT3 target gene expression (ICAM1, CCL2, IL6, SOCS3), loss of podocyte markers, recruitment, and activation of parietal epithelial cells, and, ultimately, cellular crescent formation with progressive proteinuria and renal dysfunction [79]. In anti-GBM-associated rapidly progressive glomerulonephritis, podocytes exhibit strong phosphorylation of EGFR and STAT3; glucocorticoids suppress this activation, reduce proteinuria and crescent formation, and attenuate parietal epithelial cell activation and proliferation [84]. Thus, pathological EGFR–JAK/STAT3 signaling in podocytes is most convincingly demonstrated in rapidly progressive forms of glomerulonephritis, particularly nephrotoxic serum-induced nephritis and anti-GBM-associated disease [79,84].

#### 2.2.2. Regulation and Functional Outputs of JAK–STAT Signaling in Podocytes

Canonical JAK-STAT signal transduction proceeds through cytokine-induced receptor dimerization, JAK trans-phosphorylation, and STAT recruitment and phosphorylation, followed by STAT dimerization and nuclear translocation to regulate target gene transcription. The set of activated STATs is determined by the receptor complex rather than individual JAKs: type I interferon receptors typically use JAK1/TYK2 to activate STAT1/STAT2; IFN-γ receptors signal via JAK1/JAK2 and primarily activate STAT1; IL-6/gp130-family receptors use JAK1 with JAK2 or TYK2 to mainly activate STAT3. Common γ-chain cytokine receptors, including those for IL-2, IL-7, IL-15, and IL-21, typically signal through JAK1/JAK3 and activate STAT5, with cytokine-specific contributions from other STAT proteins. Functionally, STAT1 is often associated with interferon-driven, pro-apoptotic programs, whereas STAT3 promotes expression of inflammatory, survival, and proliferative genes [76].

Direct data in podocytes most strongly support an IL-6/gp130–JAK2–STAT3 axis. Under high glucose, cultured podocytes show increased JAK2 and STAT3 phosphorylation, and neutralizing IL-6 suppresses this response and reduces cellular hypertrophy [78]. In primary glomerular epithelial cells, IL-6 and EGF induce STAT3 phosphorylation and upregulate STAT3 targets ICAM1, CCL2, IL6, and SOCS3; podocyte-specific *Stat3* deletion abolishes this response [79]. Independently, STAT1 has been shown to contribute to high-glucose-induced podocyte injury. *Stat1* silencing reduced reactive oxygen species (ROS) production, BAX abundance, and cleaved caspase-3 levels, while restoring BCL-2, FOXO1, and antioxidant defenses, including catalase, manganese superoxide dismutase, NAD(P)H quinone dehydrogenase 1, and heme oxygenase-1 [87]. Thus, direct podocyte evidence most strongly supports pathogenic IL-6/gp130–JAK2–STAT3 signaling, with an additional context-dependent role for STAT1 in high-glucose-induced oxidative injury; direct evidence for JAK3 function in mature podocytes remains limited [76,78,79,87].

Cytokine-driven JAK–STAT activation is also relevant to APOL1-associated podocytopathy. In primary human podocytes, glomerular endothelial cells, and APOL1 high-risk human kidney micro-organoids, cytokines induced by severe COVID-19 synergistically increased *APOL1* expression through JAK/STAT signaling. Pharmacological JAK1/2 inhibition with baricitinib suppressed cytokine-induced *APOL1* expression and rescued organoid podocyte viability, providing preclinical evidence that JAK–STAT signaling can couple inflammatory cytokine exposure to APOL1-dependent podocyte injury [88].

JAK–STAT signaling is negatively regulated by several mechanisms, including receptor and JAK dephosphorylation, restriction of STAT nuclear activity, and endogenous inhibitors of the SOCS and PIAS families [76]. For IL-6/gp130-family signaling, SOCS3 is a major inducible negative-feedback inhibitor. SOCS3 binds selected cytokine receptor–JAK complexes, including gp130-associated complexes, and limits sustained JAK–STAT activation [89]. In primary glomerular epithelial cells, IL-6 induces *Socs3* together with *Icam1*, *Ccl2*, and *Il6*, consistent with activation of a SOCS3-associated negative-feedback program in podocytes [79]. However, the functional requirement for SOCS3-mediated feedback in podocyte injury remains to be defined by podocyte-specific loss-of-function and rescue studies. PIAS proteins act predominantly in the nucleus and can restrain STAT-dependent transcription by modulating STAT DNA binding, transcriptional-complex assembly, and post-translational regulation [76]. Their functions in podocytes remain substantially less defined than cytokine-induced SOCS3 expression. Overall, IL-6/gp130–JAK2–STAT3 signaling represents the best-defined pathogenic JAK–STAT module in podocytes, particularly during high-glucose exposure and nephrotoxic serum-induced crescentic glomerulonephritis. STAT1 can additionally contribute to high-glucose-associated oxidative injury, whereas cytokine-induced JAK/STAT activation may promote APOL1-dependent podocyte injury in genetically susceptible settings [78,79,87,88]. The functional contributions of JAK3, TYK2, PIAS proteins, and SOCS3-mediated negative feedback require further investigation using podocyte-specific loss-of-function and rescue models [76,79,89].

#### 2.2.3. JAK–STAT Signaling in Glomerular Pathologies and In Vivo Genetic Models

JAK–STAT pathway activation is a prominent feature of diabetic kidney disease. Transcriptomic analyses of kidney biopsies from patients with early diabetic kidney disease, low-grade albuminuria, and preserved renal function identified increased expression of *JAK1*, *JAK2*, *JAK3*, *STAT1*, and *STAT3* predominantly in the glomerular compartment. In progressive diabetic kidney disease with declining renal function, JAK–STAT pathway upregulation becomes more prominent in the tubulointerstitial compartment, indicating a disease-stage-dependent shift in the dominant renal compartment in which this transcriptional program is detected [12,90].

IL-6 is an important inflammatory mediator in diabetic kidney disease. In cultured podocytes, high glucose induces a local autocrine IL-6/gp130/JAK2/STAT3 circuit, increasing IL-6 production, gp130–JAK2 interaction, JAK2 and STAT3 phosphorylation, and cellular hypertrophy; IL-6 neutralization attenuates these responses [78]. In inflammatory glomerular injury, STAT3 activation can additionally induce pro-inflammatory transcriptional programs, including *Icam1*, *Ccl2*, and *Il6*, as demonstrated in nephrotoxic nephritis models [79].

Commonly used diabetic mouse models do not fully reproduce the magnitude and compartmental pattern of JAK–STAT activation observed in progressive human diabetic kidney disease. In particular, *db/db* C57BLKS/J mice and STZ-induced diabetes in DBA/2J mice do not recapitulate the pronounced relative shift from glomerular to tubulointerstitial JAK–STAT pathway activation reported in progressive human disease [90]. By contrast, podocyte-specific JAK2 overexpression in diabetic 129S6 mice markedly worsened albuminuria, mesangial expansion, glomerulosclerosis, fibronectin accumulation, GBM thickening, and podocyte loss. Short-term systemic JAK1/2 inhibition partially reversed structural and transcriptional abnormalities, including increased STAT3 phosphorylation. These findings support a causal contribution of enhanced podocyte JAK2 signaling to diabetic glomerular injury, while not establishing a podocyte-exclusive or STAT3-exclusive pharmacological mechanism [12].

In FSGS, kidney biopsies show JAK–STAT activation, with increased phospho-STAT1 and phospho-STAT3 in glomeruli, including podocytes, and JAK–STAT gene signatures that correlate with disease severity [82]. Experimental models likewise support a role for JAK–STAT signaling in podocyte injury in vivo, although the evidence is uneven across pathway branches. In puromycin aminonucleoside nephrosis, glomerular total STAT3 and phospho-STAT3 levels increase during the development of proteinuria, indicating activation of STAT3-associated programs at the stage of active filtration-barrier injury [91].

STAT5 provides an important counterexample to the view that all JAK–STAT signaling is maladaptive in podocytes. In ADR-induced FSGS-like nephropathy, podocyte-specific *Stat5* deletion worsened albuminuria, podocyte flattening, foot-process effacement, and nephrin loss, whereas IL-15 increased podocyte STAT5 phosphorylation and attenuated glomerular injury through preservation of autophagic flux [85]. Thus, in FSGS and related nephrotic injury models, the best-supported observations are STAT1/3 activation in human biopsies, increased glomerular pSTAT3 in puromycin aminonucleoside nephrosis, and a protective role for podocyte STAT5 in ADR nephropathy. Direct podocyte-specific loss-of-function evidence for JAK2 and STAT1 in injured adult podocytes remains limited. Consequently, their cell-autonomous causal roles are less well defined than those of STAT3 and STAT5.

Beyond cytokine receptors, EGF-family ligands can amplify inflammatory podocyte responses through EGFR-associated STAT3 activation. Their pathological role is most clearly demonstrated in rapidly progressive glomerulonephritis rather than in FSGS. In nephrotoxic serum-induced crescentic glomerulonephritis, podocyte-specific *Stat3* deletion reduces proteinuria, renal functional decline, crescent formation, glomerular proliferation, loss of podocyte markers, and expression of inflammatory STAT3 target genes [79]. In anti-GBM-associated rapidly progressive glomerulonephritis, increased phospho-EGFR and phospho-STAT3 have been detected in podocytes in human samples and rat models. Glucocorticoid treatment attenuates this activation, proteinuria, crescent formation, and parietal epithelial cell activation and proliferation [84]. In cultured podocytes, HB-EGF activates EGFR/STAT3 signaling, and conditioned medium from stimulated podocytes promotes Notch-dependent proliferation of parietal epithelial cells, supporting a pathogenic podocyte–parietal epithelial cell interaction [84].

In lupus nephritis, direct evidence for podocyte-intrinsic JAK–STAT signaling has historically been more limited than in rapidly progressive glomerulonephritis but has recently expanded. IL-23R expression is increased in glomeruli from patients with lupus nephritis, and IL-23 exposure induces IL-23R expression, actin-cytoskeletal remodeling, increased motility, and STAT3 phosphorylation in podocytes. Importantly, podocyte-specific *Il23r* deletion in lupus-prone MRL/lpr mice reduces clinical and histological manifestations of lupus nephritis, providing direct genetic evidence that podocyte IL-23R signaling contributes to disease pathogenesis [92]. Systemic STAT3 signaling also contributes to lupus nephritis, as T cell-specific *Stat3* deletion in lupus-prone mice markedly attenuates nephritis [93].

Podocyte-specific genetic studies in nephrotoxic serum-induced crescentic glomerulonephritis and HIV-associated nephropathy provide the strongest direct evidence that excessive STAT3 activity can drive podocyte injury in distinct inflammatory and viral disease contexts [79,94]. In Tg26 HIV-transgenic mice, podocyte-specific *Stat3* deletion reduces albuminuria, renal functional impairment, glomerular collapse and sclerosis, podocyte proliferation and dedifferentiation, pro-inflammatory STAT3 target-gene expression, and secondary tubulointerstitial injury [94]. Consistent with this genetic evidence, the HIV-1 protein Nef activates Src-dependent STAT3 and MAPK1/2 signaling in cultured podocytes and promotes their proliferation and dedifferentiation [95].

The available genetic models that define JAK–STAT pathway functions in podocytes and glomerular disease are summarized in Table 2. Among these models, STAT3 has been investigated most extensively. Germline *Stat3* deletion causes early embryonic lethality at E6.5–E7.5 and is therefore incompatible with the analysis of adult kidney injury [96]. Tissue-specific models are consequently essential for distinguishing cell-autonomous podocyte functions from systemic effects.

Germline *Jak2* deletion causes embryonic lethality at approximately E12.5 because of severe anemia and failure of definitive erythropoiesis, precluding the analysis of adult renal phenotypes [97,98]. In diabetic kidney disease, podocyte-specific JAK2 overexpression provides the most persuasive causal genetic evidence for a pathogenic podocyte JAK2 axis, whereas the partial protection afforded by systemic JAK1/2 inhibition does not establish a podocyte-exclusive pharmacological mechanism [12]. In lupus-prone MRL/lpr mice, global *Stat1* deletion reduces proteinuria and glomerulonephritis severity but aggravates Th17-associated tubulointerstitial inflammation, accompanied by a shift toward STAT3/STAT4 signaling. Thus, the STAT1 function in the kidney is not uniformly pro-inflammatory but is compartment- and disease-context dependent [99].

Overall, the pathogenic role of podocyte STAT3 is supported most directly by podocyte-specific genetic studies in nephrotoxic nephritis and HIV-associated nephropathy. In diabetic glomerular injury, the available evidence most strongly supports a pathogenic podocyte JAK2–STAT3 axis, but direct podocyte-specific *Stat3* loss-of-function studies remain necessary. By contrast, STAT5 preserves podocyte integrity in ADR-induced FSGS-like injury, whereas STAT1 exerts compartment- and disease-dependent effects in lupus nephritis. These findings indicate that therapeutic modulation of JAK–STAT signaling should preserve adaptive STAT-dependent survival programs while selectively limiting context-specific pathogenic signaling.

#### 2.2.4. Epitranscriptomic Regulation of Inflammatory Podocyte Responses

Beyond classical kinase and cytokine signaling, podocyte responses to inflammatory and metabolic stress are increasingly recognized to be shaped by epitranscriptomic mechanisms, including RNA N^6^-methyladenosine (m^6^A) modification. Among RNA m^6^A writer complexes, METTL14 has emerged as a direct regulator of podocyte injury. METTL14 expression and global m^6^A RNA abundance were increased in ADR nephropathy, diabetic nephropathy, and human FSGS or diabetic nephropathy biopsy samples, and METTL14 was increased in injured podocytes. In ADR nephropathy, podocyte-specific *Mettl14* deletion improved glomerular function and attenuated podocyte injury, with enhanced autophagy and reduced apoptosis and inflammatory responses. In cultured podocytes exposed to ADR or advanced glycation end products, METTL14 knockdown similarly promoted autophagy and reduced apoptosis and inflammatory responses. Mechanistically, METTL14 promoted m^6^A modification and degradation of *Sirt1* mRNA, whereas METTL14 suppression restored SIRT1 expression in injured podocytes [100].

A related, but cell-distinct, mechanism has been described in glomerular endothelial cells. In high-glucose-exposed human renal glomerular endothelial cells, METTL14 increased ROS, TNF, IL-6, and apoptosis while reducing α-Klotho expression through an m^6^A-dependent mechanism. In *db/db* mice, systemic rAAV-mediated METTL14 overexpression aggravated renal injury and inflammatory indices, whereas α-Klotho overexpression partially ameliorated these effects. Thus, METTL14-dependent RNA methylation may amplify stress responses in both podocytes and glomerular endothelial cells; however, the endothelial study does not establish a podocyte-autonomous mechanism [101].

Together, these findings identify m^6^A-dependent post-transcriptional regulation as an additional layer of control over podocyte stress responses and support the investigation of cell-selective epitranscriptomic interventions in proteinuric glomerular disease. Whether METTL14 directly integrates cytokine-induced JAK–STAT or NF-κB signaling with podocyte m^6^A remodeling remains to be established.

JAK–STAT signaling should therefore be interpreted at the level of individual cytokine–STAT circuits rather than as a uniformly pathogenic pathway. The balance between injury and adaptation depends on the STAT protein, cellular context, and disease model. Human pathway associations establish relevance but do not alone demonstrate podocyte-specific causality.

### 2.3. Cytoskeletal Integration: Rho-GTPases

RhoA, Rac1, and Cdc42 are considered here primarily as spatially regulated cytoskeletal and mechanotransduction integrators rather than dedicated inflammatory sensors. Their coupling to inflammatory pathways can be bidirectional, but the strength of direct evidence differs among individual signaling relationships. The Rho family of small GTPases, principally RhoA, Rac1, and Cdc42, functions as molecular switches that cycle between active GTP-bound and inactive GDP-bound states to dynamically regulate the actin cytoskeleton, cell shape, motility, and adhesion [102]. Dysregulation of RhoA/Rac1/Cdc42 activity is increasingly recognized as a common final pathway in hereditary and acquired podocytopathies [103,104,105,106]. In podocytes, Rho-GTPases serve as central integrators of signals from slit diaphragm proteins, mechanical stretch, and pro-inflammatory pathways, including NF-κB, JAK-STAT, and p38 MAPK [104,107]. These inflammatory pathways cause structural dysfunction through Rho-GTPase-dependent actin remodeling, resulting in foot process effacement and barrier loss.

#### 2.3.1. Activation, Regulation, and Function of RhoA/Rac1/Cdc42 in Podocytes

In podocytes, Rho-GTPases are activated in response to a broad range of extracellular stimuli, including mechanical stretch, high glucose, TGF-β, angiotensin II, glucocorticoids, anti-nephrin antibodies, and ligands for slit diaphragm proteins and integrins [108].

Classical studies established that nephrin/podocin and TRPC6 transmit signals to the actin cytoskeleton through Rho-GTPases and their regulatory proteins (guanine nucleotide exchange factors GEFs, GTPase-activating proteins GAPs, and GDP-dissociation inhibitors GDIs), thereby controlling foot process dynamics [107]. An additional level of spatial control is provided by lipid rafts, specialized plasma-membrane microdomains enriched in cholesterol and sphingolipids that organize slit-diaphragm-associated signaling complexes. In podocytes, raft-associated membrane organization supports the localization and function of nephrin, podocin, and TRPC6. Because TRPC6-dependent Ca^2+^ signaling interfaces with Rho GTPase-controlled actin remodeling, alterations in raft composition may influence cytoskeletal homeostasis and filtration-barrier integrity [109,110,111].

RhoGDIα retains Rho-family GTPases in an inactive cytosolic pool and thereby limits inappropriate signaling. In RhoGDIα-deficient mice, LPS exposure induces more severe proteinuria and podocyte injury than in wild-type animals. Complementary studies in cultured podocytes show that loss of RhoGDIα enhances Rho-family GTPase activity and alters actin-cytoskeletal dynamics, supporting a podocyte-intrinsic buffering role for RhoGDIα while not establishing that the in vivo phenotype is exclusively podocyte autonomous [112].

GEFs promote nucleotide exchange and activation of Rho-family GTPases, whereas GAPs terminate signaling by accelerating GTP hydrolysis. ARHGEF12 has emerged as a candidate regulator of RhoA in podocytes, whereas SRGAP2a and SRGAP1 provide distinct GAP-mediated restraints on cytoskeletal remodeling. In diabetic nephropathy, SRGAP2a is downregulated in podocytes; high glucose and TGF-β suppress its expression, whereas SRGAP2a limits podocyte motility by inactivating RhoA and Cdc42. In parallel, SRGAP1 is a podocyte-enriched RhoGAP that constrains excessive protrusive actin-network activity required for foot-process maintenance. Together, these observations indicate that the fine-tuned spatial and temporal control of RhoA, Rac1, and Cdc42 activity is essential for preserving podocyte architecture and homeostasis [113,114].

A BioID-based proximity proteomic analysis of human podocytes identified 1927 proximity interactors of RhoA, Rac1, and Cdc42, approximately half of which were unique to podocytes relative to HEK293 and HeLa cells and were enriched in pathways related to cell adhesion and shape organization. This analysis identified KIAA1522 as a novel Cdc42 effector and ARHGEF12 as a key regulator of RhoA, expanding the candidate network through which Rho-family GTPases control podocyte architecture [115].

RhoA, Rac1, and Cdc42 play partially overlapping but functionally divergent roles in podocytes. RhoA, through its effector kinases ROCK1/2 and formins such as mDia, contributes to stress-fiber organization and foot-process structural integrity. Both reduced and sustained excessive RhoA activity can disrupt cytoskeletal homeostasis. In inducible podocyte-specific transgenic mice, constitutively active or dominant-negative RhoA induces albuminuria and foot-process injury. Complementary studies in cultured podocytes show that RhoA depletion disrupts actin stress fibers, reduces YAP expression, and increases apoptosis through a dendrin-dependent mechanism [103,116].

Rac1 and Cdc42 regulate lamellipodia and filopodia formation, podocyte motility, and adaptive cytoskeletal reorganization. Cdc42 is indispensable for the establishment and maintenance of baseline podocyte architecture: podocyte-specific *Cdc42* deletion causes congenital nephropathy with severe neonatal proteinuria, diffuse foot-process effacement, altered podocyte polarity, mislocalization of nephrin and podocin, collapsing glomerular lesions, and early lethality. By contrast, podocyte-specific deletion of *Rac1* or *RhoA* causes little overt baseline glomerular injury in the same developmental setting, although both pathways are important in stimulus- and disease-dependent injury responses [117,118].

#### 2.3.2. Cross-Talk with NF-κB, p38 MAPK, and JAK-STAT

Rho-family GTPase signaling is tightly integrated with other intrinsic inflammatory pathways discussed in this review. RhoA/ROCK activity intersects with NF-κB-associated inflammatory signaling, creating bidirectional links between cytoskeletal stress, oxidative injury, and inflammatory gene expression. In mechanically stressed podocytes, Rac1 and RhoA activation promoted a TGF-β-dependent mesenchymal-like phenotypic shift, whereas pharmacological Rac1 or ROCK inhibition attenuated albuminuria and glomerular structural injury in 5/6 nephrectomy models [108]. In diabetic podocytopathy, fasudil reduced albuminuria, podocyte apoptosis, and Jag1/Notch-associated pro-apoptotic signaling, supporting a role for ROCK activity in coupling TGF-β-associated stress to podocyte injury [119]. Conversely, NF-κB-dependent inflammatory programs may reciprocally influence the expression and activity of cytoskeletal regulators, although the precise directionality and cell-autonomous mechanisms of this feedback remain incompletely defined.

Stress-activated MAPKs, particularly p38, provide another interface between inflammatory signaling and the podocyte cytoskeleton. In diabetic podocytes, loss of the phosphatase DUSP4 sustained p38 and JNK activation and was associated with increased NOX4 expression, caspase-3/7 activity, podocyte death, and foot-process injury [71]. Sustained Rac1 hyperactivation also induced podocyte detachment and FSGS-like glomerulosclerosis through a mechanism that was partially dependent on p38 MAPK activity [120]. These observations support a p38–Rho-family GTPase nexus in which stress-activated inflammatory signaling can be translated into cytoskeletal destabilization, loss of slit-diaphragm integrity, and progressive podocyte depletion. However, the consequences of p38 loss are highly context-dependent. In the setting of combined podocyte-specific GC-A and p38 MAPK deletion, aldosterone/high-salt stress aggravated podocyte and endothelial injury and caused glomerular intra-capillary thrombosis [68].

JAK–STAT signaling may also influence podocyte cytoskeletal homeostasis, although direct mechanistic evidence linking this pathway to individual Rho-family GTPases remains less mature. In cultured podocytes lacking the neonatal Fc receptor, impaired IL-6 production and reduced STAT3 phosphorylation in response to inflammatory or immune-complex stimuli were accompanied by abnormal stress fibers, altered motility, and reduced synaptopodin expression. Restoration of IL-6 partially rescued the stress-fibre phenotype, supporting a role for IL-6–STAT3 signaling in actin-cytoskeletal homeostasis [121]. CLCF1 activates STAT3 and induces rapid, concentration-dependent actin-cytoskeletal remodeling in cultured podocytes, characterized by loss of ordered basal stress fibers and expansion of lamellipodia [83]. In high-glucose-exposed podocytes, local IL-6/gp130/JAK2/STAT3 activation promotes cellular hypertrophy; whether this pathway directly regulates Rho-family GTPase activity in diabetic podocytes remains to be resolved [78]. In lupus-prone *MRL/lpr* mice, fasudil preserved podocyte actin architecture in association with CaMK4/Rho-family GTPase–YAP signaling, although concurrent effects on systemic autoimmunity and immune-complex deposition preclude attribution of this response exclusively to podocyte-autonomous mechanisms [122]. Thus, compared with the p38–Rho-family GTPase and NF-κB–Rho-family GTPase axes, direct JAK–STAT–Rho-family GTPase coupling in podocytes remains incompletely defined.

#### 2.3.3. Rho-GTPase Dysregulation in Proteinuric Kidney Diseases

In diabetic kidney disease, metabolic stress, TGF-β signaling, and oxidative injury can disrupt Rho-family GTPase homeostasis, promoting maladaptive actin remodeling, loss of slit-diaphragm integrity, podocyte apoptosis, and detachment. The contribution of individual pathway components is not uniform: excessive ROCK signaling is pathogenic in several diabetic models, whereas basal RhoA, Rac1, and Cdc42 activity can support cytoskeletal adaptation and survival. Sustained Rac1 activation is likewise injurious in experimental podocytopathies, although its role in diabetic podocyte injury remains context dependent. In cultured podocytes exposed to LPS or ADR, siRNA-mediated depletion of RhoA or mDia disrupted actin stress fibers, reduced YAP expression, and promoted dendrin-dependent apoptosis. In *db/db* mice, systemic Rho-kinase inhibition with fasudil reduced albuminuria, urinary nephrin excretion, and podocyte apoptosis, accompanied by suppression of a TGF-β/ROCK–Jag1/Notch-associated pro-apoptotic program [119]. Genetic evidence further identifies ROCK2 as a pathogenic mediator of diabetic podocytopathy: podocyte-specific *Rock2* deletion protected against albuminuria, podocyte loss, glomerular structural injury, and fibrosis while restoring PPARα-dependent fatty-acid oxidation [123].

In lupus nephritis, fasudil preserved nephrin and synaptopodin expression, reduced podocyte apoptosis, and improved actin cytoskeletal integrity in lupus-prone *MRL/lpr* mice. These effects were associated with altered CaMK4/Rho-family GTPase signaling and restoration of nuclear YAP activity; however, concurrent reductions in systemic autoimmunity and glomerular immune-complex deposition preclude attribution of the in vivo benefit exclusively to podocyte-autonomous ROCK inhibition [122]. In anti-nephrin antibody-induced nephropathy, anti-nephrin antibodies induce TRPC6-dependent Ca^2+^ influx and phosphorylation of nephrin and ephrin-B1, promoting release of Par6, stabilization of Cdc42, and increased Cdc42 activity. This pathway disrupts podocyte polarity and initiates proteinuria, indicating that Cdc42 hyperactivation can be pathogenic in a disease-specific setting [124]. Conversely, synaptopodin protects the filtration barrier by disrupting Cdc42–IRSp53–Mena signaling complexes and limiting aberrant Cdc42-dependent filopodia formation in podocytes [125].

In progressive chronic kidney disease, mechanical stretch activates Rac1 and RhoA in podocytes and induces a TGF-β-dependent mesenchymal-like phenotypic shift characterized by reduced WT1 and nephrin expression and increased α-SMA and fibronectin expression. In a 5/6 nephrectomy model, systemic Rac1 or ROCK inhibition attenuated albuminuria and structural glomerular injury, supporting a role for Rho-family GTPase signaling in mechanically driven podocyte dysfunction while not establishing a podocyte-exclusive in vivo mechanism [108].

The distinct and context-dependent functions of individual Rho-family GTPases are most clearly demonstrated by podocyte-specific genetic models, which are summarized in Table 3. Podocyte-specific *Cdc42* deletion causes congenital nephropathy with severe proteinuria, diffuse foot-process effacement, loss of podocyte polarity, nephrin and podocin mislocalization, collapsing glomerular lesions, and early lethality, whereas podocyte-specific *Rac1* and *RhoA* deletion produces little overt baseline injury [117,126]. These findings identify Cdc42 as indispensable for baseline podocyte polarity and architecture. By contrast, the effects of Rac1 are injury-dependent: podocyte-specific Rac1 deletion reduces acute protamine sulfate-induced foot-process effacement but aggravates chronic hypertensive injury and ADR-associated glomerulosclerosis, indicating a role for basal Rac1 signaling in selected repair and adaptive responses [118,126].

Conversely, persistent activation of Rac1 or RhoA is pathogenic. Inducible constitutively active Rac1 expression in podocytes causes a dose-dependent spectrum from proteinuria and foot-process effacement to podocyturia, podocyte detachment, and FSGS-like glomerulosclerosis, with detachment partially dependent on p38 MAPK activity [120]. Similarly, inducible RhoA activation causes albuminuria, synaptopodin loss, and FSGS-like injury, demonstrating that podocyte integrity depends on dynamically regulated rather than constitutively high RhoA activity [127].

Collectively, these findings position Rho-family GTPases and their regulatory networks as central determinants of podocyte polarity, cytoskeletal adaptation, focal-adhesion turnover, and injury responses. Cdc42 is required for basal podocyte architecture, whereas Rac1, RhoA, ARF6, and ROCK2 exert context-, dose-, and time-dependent effects. Regulators including RhoGDIα, SRGAP proteins, and ROCK isoforms further demonstrate that precise spatial and temporal control of Rho-family GTPase activity is required to preserve foot processes and filtration-barrier integrity [114,123,128,129,130]. This signaling module intersects with NF-κB, p38 MAPK, and JAK–STAT pathways and provides a mechanistic bridge between extracellular inflammatory or mechanical stress and the intracellular sensors of mitochondrial and nucleic-acid damage discussed in the following section.

**Table 3:** Genetic perturbation models defining Rho-family GTPase and ROCK functions in podocytes.

Mediator	Genetic Manipulation	Experimental Model or Context	Principal Phenotype	Reference
Cdc42	Podocyte-specific conditional deletion	Development and spontaneous phenotype	Severe congenital proteinuria, diffuse foot-process effacement, loss of podocyte polarity, nephrin/podocin mislocalization, progressive glomerular injury, and early lethality	[117,126]
Rac1	Podocyte-specific conditional deletion	Baseline; protamine sulfate injury; chronic hypertensive injury	Minimal baseline phenotype; reduced acute foot-process effacement after protamine sulfate exposure; increased albuminuria and glomerulosclerosis during chronic hypertensive injury	[126]
Rac1	Podocyte-specific conditional deletion	ADR nephropathy	Increased podocyte detachment and glomerulosclerosis, with reduced mTOR-associated podocyte hypertrophy during repair	[118]
Rac1	Inducible podocyte-specific constitutively active expression	Dose-dependent nephrotic syndrome model	Foot-process effacement and proteinuria at lower Rac1 activation; podocyturia, podocyte detachment, and FSGS-like glomerulosclerosis at higher or sustained activation	[120]
RhoA	Podocyte-specific conditional deletion	Baseline/developmental homeostasis	No overt baseline proteinuria or major structural phenotype, indicating that RhoA is less essential than Cdc42 for baseline podocyte homeostasis in this developmental model	[117]
RhoA	Inducible podocyte-specific constitutively active expression	Doxycycline-inducible transgenic podocytopathy	Dose-dependent albuminuria and foot-process effacement; low-level activation caused segmental foot-process injury, whereas higher or prolonged activation induced extensive foot-process effacement and FSGS-like glomerulosclerosis	[127]
ARF6	Podocyte-specific conditional deletion	Protamine sulfate-induced acute injury; nephrotoxic serum nephritis	No overt baseline proteinuria or major structural phenotype. Protection from foot-process effacement after protamine sulfate injury but increased proteinuria and more severe glomerular injury in nephrotoxic serum nephritis, supporting a context-dependent role of ARF6 in nephrin-associated podocyte dynamics.	[131]
ROCK2	Podocyte-specific conditional deletion	ADR-induced FSGS-like nephropathy	Reduced albuminuria, glomerulosclerosis, podocyte loss, foot-process widening, GBM thickening, and tubulointerstitial fibrosis	[130]
ROCK2	Podocyte-specific conditional deletion	Diabetic models	Reduced albuminuria, podocyte loss, and glomerular fibrosis; restoration of PPARα-dependent fatty-acid oxidation	[123]
RhoGDIα	Global RhoGDIα deficiency; complementary loss-of-function studies in cultured podocytes	Baseline and LPS-associated podocyte injury	Global RhoGDIα deficiency increased susceptibility to LPS-associated proteinuria and podocyte injury. In cultured podocytes, RhoGDIα loss enhanced Rho-family GTPase activity and disrupted actin-cytoskeletal dynamics; the in vivo phenotype cannot be assigned exclusively to podocytes.	[112]
RhoGDIα/*ARHGDIA*	Human homozygous loss-of-function mutation and complementary experimental models	Hereditary nephrotic syndrome	Loss of RhoGDIα disrupts normal regulation of Rac1, RhoA, and Cdc42, leading to impaired cell motility, actin-cytoskeletal dysregulation, and nephrotic syndrome	[128,129]

Note: Podocyte-restricted genetic manipulation supports a contribution of the targeted pathway within podocytes but does not, by itself, establish an adult-specific function or independence from intercellular signaling. Global genetic manipulation does not establish podocyte specificity; developmental timing and inducibility should be considered when interpreting each model.

### 2.4. Cytosolic Nucleic-Acid Sensing, Inflammasome Activation

Podocytes harbor an intracellular nucleic acid surveillance system capable of initiating inflammatory cascades in response to sterile danger signals. The cGAS–STING pathway, which detects cytosolic double-stranded DNA (dsDNA), represents one of the most interesting intrinsic inflammatory axes in podocytes and exhibits several specific features that distinguish it from innate immune cells [132,133]. ZBP1 is an interferon-inducible Z-nucleic-acid sensor that can engage RIPK3-dependent necroptotic signaling and participate in PANoptosome-mediated inflammatory cell death, including PANoptosis, during sustained innate immune activation. Most mechanistic evidence for these functions derives from viral and other infectious disease models. Whether a cGAS–STING–ZBP1 axis directly operates in podocytes remains to be defined [134,135]. The NLRP3 inflammasome functions as a convergent effector platform that integrates signals from multiple pathways, including NF-κB, cGAS–STING, and metabolic stress. Its activation drives caspase-1-dependent cytokine maturation and, when coupled to gasdermin-mediated membrane pore formation, culminates in pyroptosis—a highly destructive form of podocyte death. In this section, we focus on cytosolic DNA sensing in podocytes, the role of NLRP3 in glomerular diseases, and ZBP1 as an amplifier of podocyte cell death.

#### 2.4.1. Cytosolic DNA Sensing in Podocytes

Activation of the cGAS–STING pathway proceeds through sequential steps involving cytosolic dsDNA recognition by cGAS, synthesis of 2′3′-cGAMP, STING activation and trafficking, and engagement of downstream TBK1/IRF3- and NF-κB-dependent effector programs [133]. Cyclic GMP–AMP synthase (cGAS) recognizes cytosolic dsDNA largely independently of nucleotide sequence but in a DNA-length-dependent manner. Short dsDNA fragments can support cGAS activation, whereas longer DNA molecules more efficiently promote cGAS oligomerization and 2′3′-cGAMP production [136].

cGAS can be activated not only by pathogen-derived DNA from viruses, bacteria, and parasites but also by host-derived nuclear or mitochondrial DNA that reaches the cytosol during genomic instability, mitochondrial dysfunction, cellular senescence, and selected forms of regulated cell death [132,137]. Genome instability can generate micronuclei and other extranuclear chromatin structures. Following micronuclear-envelope rupture, self-DNA may become accessible to cGAS and initiate a cell-intrinsic inflammatory response; however, the magnitude and consequences of this response depend on chromatin context and cell type [138]. cGAS is also present in the nucleus, where its binding to nucleosomes restrains inappropriate activation by chromatinized self-DNA. Nucleosome-associated cGAS engages the acidic patch of histones H2A–H2B and is maintained in an inactive conformation that limits dsDNA-dependent dimerization and cGAMP synthesis [139].

Whether nuclear cGAS sequestration, micronuclear rupture, or chromatin-bridge formation contributes directly to cGAS–STING activation in injured podocytes remains insufficiently defined. In contrast, mitochondrial DNA leakage during metabolic and lipotoxic stress has been directly demonstrated as a source of cytosolic DNA that activates cGAS–STING signaling in podocytes. Other potential sources of cytosolic DNA, including NET-derived extracellular DNA in lupus nephritis and LINE-1-derived reverse-transcribed nucleic acids during cellular senescence, are biologically plausible but have not yet been established as podocyte-autonomous cGAS–STING triggers [132].

#### 2.4.2. Predominance of NF-κB-Associated Output over IRF3 Signaling in Podocytes

Following dsDNA recognition, activated cGAS synthesizes the cyclic dinucleotide 2′3′-cGAMP, which binds to the ER-resident transmembrane adaptor STING and promotes its conformational activation and trafficking from the ER to the Golgi. At the Golgi, STING recruits and activates TBK1. TBK1 phosphorylates STING, generating a phospho-dependent docking site for IRF3; recruited IRF3 is then phosphorylated by TBK1, dimerizes, and translocates to the nucleus to induce type I interferon and interferon-stimulated gene expression [140,141].

The STING–TBK1–IRF3 module induces type I interferons and a context-dependent interferon-stimulated gene program that can include *IFIT1*, *IFIT2*, *IFIT3*, *MX1*, *CXCL10*, and *IRF7* [142]. ZBP1 is an interferon-inducible Z-nucleic-acid sensor that can couple sustained innate immune activation to inflammatory cell-death programs; however, its direct contribution downstream of cGAS–STING in injured podocytes remains to be established [134,135,143]. In parallel with IRF3 activation, STING can engage TRAF6- and IKK-dependent mechanisms that promote NF-κB activation and context-dependent expression of inflammatory cytokines and chemokines [144]. The contribution of TBK1 to the NF-κB branch is cell-type and stimulus dependent: in myeloid cells, TBK1 and IKKε act redundantly downstream of STING, whereas TBK1 alone is not universally required for STING-induced NF-κB activation [145,146].

In innate immune cells, cGAS–STING activation commonly induces a robust type I interferon response and a broad antiviral interferon-stimulated gene program [142]. By contrast, in podocytes, cGAS–STING activation is associated with a relatively limited IRF3/type I interferon response but robust NF-κB-associated inflammatory output, including increased TNF and IL-6 expression together with reduced nephrin and podocin abundance [132]. In cultured podocytes and diabetic or obese mouse models, metabolic and lipotoxic stress induces mitochondrial injury and BAX-dependent mtDNA leakage into the cytosol, activating cGAS–STING signaling and promoting inflammatory podocyte injury [132]. In this experimental setting, NF-κB-associated inflammatory programs and TNF/IL-6 output are more sustained than the comparatively limited IRF3/type I interferon response, supporting NF-κB-associated signaling as a dominant driver of persistent podocyte injury [132]. However, the mechanisms through which NF-κB-associated inflammatory signaling suppresses slit-diaphragm gene expression in podocytes remain incompletely defined.

#### 2.4.3. Priming and Activation of the NLRP3 Inflammasome in Podocytes

Inflammasomes are cytosolic multiprotein platforms that promote caspase-1 activation, maturation of IL-1β and IL-18, and, when gasdermin D (GSDMD) is cleaved, pyroptotic cell death. Although several inflammasomes have been described in myeloid cells, including NLRP1, NLRP3, NLRC4, and AIM2, available studies in podocytes most strongly support a role for NLRP3 [147].

cGAS–STING signaling can intersect with NLRP3 activation through several mechanisms in a cell-type- and disease-dependent manner. In non-podocyte systems, STING can directly bind NLRP3, promote its localization to the endoplasmic reticulum, and facilitate NLRP3 deubiquitination, thereby supporting inflammasome assembly [148]. In podocytes, direct evidence for functional cGAS–STING–NLRP3 coupling has been obtained in APOL1-associated podocytopathy, in which APOL1 risk variants promote mitochondrial dysfunction, cytosolic mtDNA leakage, STING and NLRP3 activation, caspase-1 activation, GSDMD cleavage, and inflammatory podocyte injury [149]. These data support a model in which cGAS–STING signaling can provide an upstream inflammatory context for NLRP3 activation in podocytes under selected disease conditions. However, the relative contributions of NF-κB-dependent priming, mitochondrial ROS, direct STING–NLRP3 interaction, and type I interferon signaling remain to be determined in individual podocytopathies. Thus, cGAS–STING and NLRP3 should be considered interconnected but not universally linear components of podocyte-intrinsic inflammatory signaling.

Canonical NLRP3 inflammasome activation is commonly described as a two-step process. A priming signal, often delivered through pattern-recognition receptors or cytokine receptors, activates NF-κB and increases the expression of *NLRP3* and *pro-IL1B*. A second signal, generated by cellular perturbations such as K^+^ efflux, mitochondrial dysfunction, ROS, lysosomal damage, or extracellular ATP, promotes inflammasome assembly, caspase-1 activation, cytokine maturation, and, when GSDMD is cleaved, pyroptotic cell death [147,150].

Podocytes express a context-dependent repertoire of innate immune and cytokine receptors that can contribute to inflammatory priming. Cultured podocytes constitutively express several cell-surface Toll-like receptors, including TLR4, and can induce additional receptors under pathological stress. For example, TLR9 is upregulated in podocytes in experimental injury and can respond to endogenous mitochondrial DNA [8,151]. TNF receptors, IL-1 receptors, RAGE, and complement receptors can also provide inflammatory inputs to podocytes, but the relative contribution of each receptor to NLRP3 priming differs among disease models and should not be assumed to be uniform [28].

In podocytes, several disease-associated stimuli may provide activation signals that converge on NLRP3-associated injury pathways. Mitochondrial dysfunction and oxidative stress are particularly relevant because podocytes require tightly regulated mitochondrial function to maintain actin-cytoskeletal organization, foot-process dynamics, and filtration-barrier integrity [152]. In angiotensin II-induced podocyte injury, NLRP3 activation is associated with mitochondrial dysfunction and podocyte damage, whereas genetic Nlrp3 deletion attenuates this phenotype [153]. Complement activation provides another disease-specific context: in membranous nephropathy, C3a and C5a stimulation induces expression of pyroptosis-associated molecules in cultured human podocytes, and inhibition of pyroptosis attenuates complement-induced podocyte injury [154].

Direct genetic evidence further supports a functional role for podocyte NLRP3 in diabetic kidney disease. Podocyte-specific expression of the gain-of-function *Nlrp3A350V* variant aggravated albuminuria, mesangial expansion, and GBM thickening in hyperglycemic mice, whereas podocyte-specific deletion of *Nlrp3* or *Casp1* provided protection. Notably, podocyte-specific *Nlrp3* deletion was more protective than *Casp1* deletion, suggesting that NLRP3 may exert both canonical inflammasome-dependent and inflammasome-independent functions in diabetic podocytopathy [155].

However, canonical NLRP3 inflammasome activity in podocytes remains controversial. Single-cell transcriptomic analyses, studies in differentiated primary human podocytes, and diabetic mice with podocyte-specific expression of the NLRP3-A350V Muckle–Wells variant found low or undetectable expression of key inflammasome components in kidney epithelial cells and no stress-induced secretion of mature IL-1β or IL-18 from podocytes. These data suggest that, at least in the examined diabetic settings, canonical NLRP3-dependent glomerular inflammation may be driven predominantly by intrarenal immune cells rather than by podocytes [156]. Thus, the contribution of NLRP3 to podocyte injury should be interpreted according to disease context, experimental system, and whether canonical caspase-1-dependent inflammasome activity or inflammasome-independent NLRP3 functions are being assessed.

#### 2.4.4. NLRP3-Associated Podocyte Injury: Evidence for Canonical Inflammasome Activity and Pyroptosis

In cells competent for canonical NLRP3 inflammasome signaling, priming and an appropriate activation stimulus promote NLRP3 conformational rearrangement and nucleotide-dependent oligomerization. NLRP3 contains an N-terminal pyrin domain (PYD), a central NACHT domain that mediates nucleotide-dependent oligomerization, and C-terminal leucine-rich repeats involved in its regulation. NEK7 participates in licensing canonical NLRP3 activation downstream of potassium efflux and supports assembly of the active complex. NLRP3 recruits the adaptor ASC through PYD–PYD interactions, enabling CARD-dependent recruitment and activation of procaspase-1. Active caspase-1 processes pro-IL-1β and pro-IL-18 and cleaves GSDMD, releasing its pore-forming N-terminal fragment [157,158,159]. GSDMD pores can facilitate cytokine release without necessarily causing immediate cell lysis, although sustained membrane permeabilization can culminate in pyroptotic cell death [157,158,159,160]. This established pathway provides a mechanistic framework, but its functional competence in podocytes remains disputed.

The podocyte literature links NLRP3-associated injury to several disease contexts. Wu et al. implicated NLRP3, STING, and GSDMD in APOL1 risk variant-associated podocytopathy [149]. Complement-mediated injury provides another relevant setting, given the multiple interactions between complement activation and podocyte stress responses. In differentiated immortalized human podocytes, Wang et al. reported that C3a and C5a stimulation increased GSDMD cleavage, membrane permeability, and lactate dehydrogenase release; pharmacological inhibition of NLRP3, caspase-1, or gasdermin-associated signaling attenuated these changes [154]. Mitochondrial dysfunction is a recurring feature of experimental podocytopathies, and Zhao et al. linked angiotensin II-induced mitochondrial abnormalities and podocyte injury to NLRP3 using cultured-cell knockdown and global Nlrp3-deficient mice [153]. However, mitochondrial stress does not uniquely identify an NLRP3-dependent mechanism: Bao et al. demonstrated an alternative pathway in which endogenous mitochondrial DNA accumulated in endolysosomes and promoted TLR9-dependent signaling and apoptosis in cultured podocytes [151]. These studies therefore need to be interpreted according to the pathway components, cellular models, and functional endpoints actually examined.

More direct evidence supporting a pathogenic role for podocyte NLRP3 comes from the gain- and loss-of-function experiments of Shahzad et al. [155]. In hyperglycemic mice, podocyte-specific expression of the Nlrp3-A350V gain-of-function variant aggravated albuminuria, mesangial expansion, and glomerular basement membrane thickening. Conversely, podocyte-specific Nlrp3 deficiency protected against diabetic glomerular injury, whereas podocyte-specific caspase-1 deficiency afforded only partial protection. Glomerular autophagy was maintained by Nlrp3 deficiency but not by caspase-1 deficiency, supporting a contribution from caspase-1-independent NLRP3 functions in addition to canonical signaling [155].

In contrast, Kunte et al. did not reproduce a pathogenic effect of podocyte-restricted Nlrp3-A350V expression in female C57BL/6J mice subjected to streptozotocin-induced diabetes and uninephrectomy [156]. Using constitutive Nphs2-Cre-mediated expression of the conditional A350V allele, they found no genotype-dependent increase in albuminuria or deterioration of slit-diaphragm density. Morphometric analysis instead showed higher podocyte counts and increased podocyte nuclear area in the mutant group. Their transcriptomic analyses also did not support appreciable expression of the canonical inflammasome machinery in podocytes. Importantly, the human in vitro experiments used renal progenitor cells differentiated into podocyte-like cells with panobinostat, rather than directly isolated mature podocytes. These cells did not secrete detectable IL-1β or IL-18 after LPS priming and ATP stimulation, whereas differentiated THP-1 cells served as a positive control [156].

**Figure 3:**
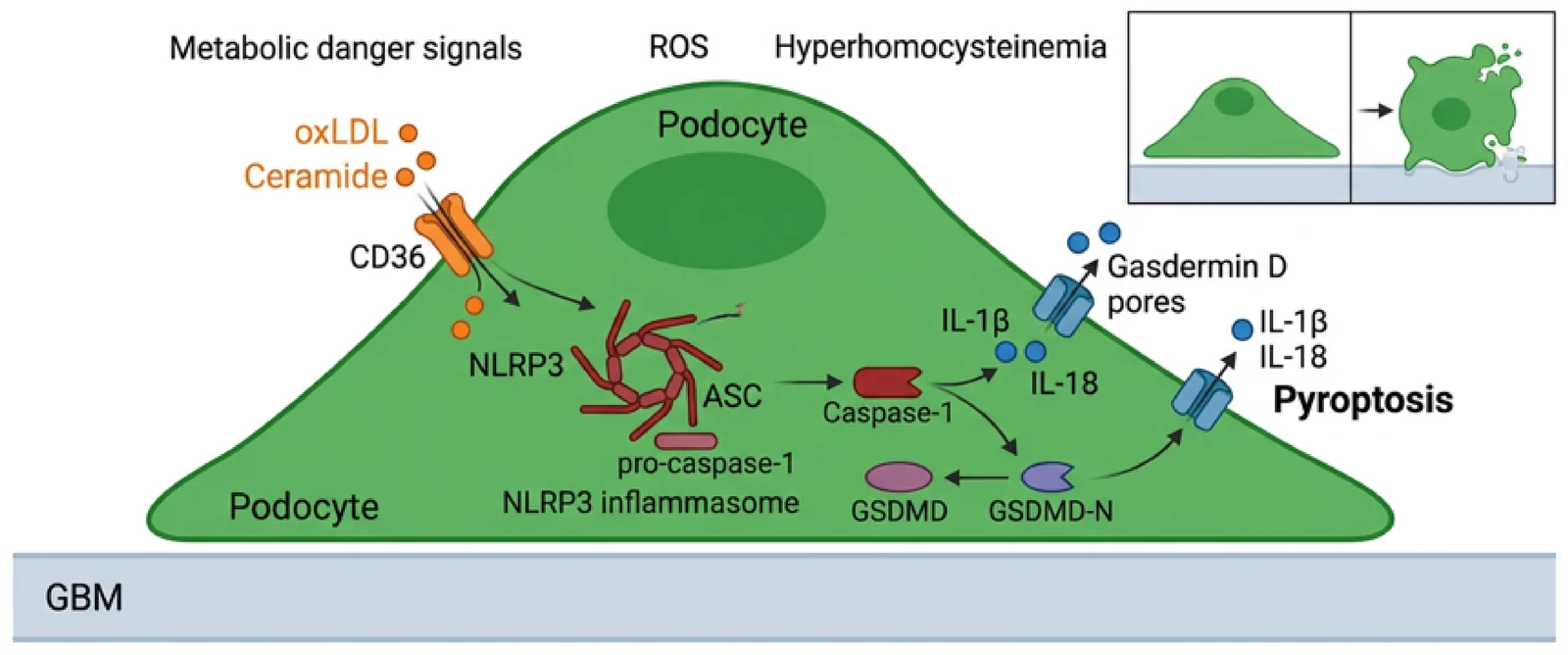
Proposed framework of NLRP3-associated signaling and pyroptotic injury in stressed podocytes. Metabolic, oxidative, and inflammatory stressors, including oxidized lipids, hyperhomocysteinemia, ROS, ceramide, extracellular ATP, and complement-associated membrane stress, are depicted as potential inputs into NLRP3-associated signaling. In cells competent for canonical inflammasome signaling, activated NLRP3 recruits ASC and procaspase-1, leading to caspase-1 activation, IL-1β and IL-18 maturation, and GSDMD cleavage. GSDMD pores can facilitate cytokine release without necessarily causing cell lysis, although membrane permeabilization can culminate in pyroptotic cell death. The proposed downstream consequences in podocytes include actin-cytoskeletal disorganization, slit-diaphragm disruption, and podocyte loss, potentially contributing to filtration-barrier dysfunction and proteinuria. However, the functional competence of the complete canonical NLRP3–ASC–caspase-1–GSDMD pathway in podocytes remains disputed. The schematic therefore integrates reported and proposed mechanisms rather than depicting a universally established sequence. Sublytic GSDMD-dependent cytokine release has been demonstrated in macrophages, but an analogous mechanism of podocyte structural dysfunction remains unproven. Podocyte-intrinsic responses should also be distinguished from inflammasome activity in resident and infiltrating immune cells.

Taken together, these studies leave an unresolved discrepancy concerning canonical inflammasome competence and the consequences of NLRP3 activity in podocytes. Differences in experimental design, cellular state, and analytical sensitivity are possible contributors, but have not been shown to explain the divergent results. Functional effects of podocyte NLRP3 should therefore be distinguished from proof of the complete NLRP3–ASC–caspase-1–GSDMD pathway [155,156,157,161,162]. NLRP3 expression, inflammasome assembly, caspase-1 activation, cytokine maturation and release, GSDMD cleavage, and lytic cell death are distinct endpoints and should not be treated as interchangeable [157,158,159,160,161]. In particular, NLRP3 activation per se does not establish pyroptotic cell death, and GSDMD pore formation does not necessarily entail cell lysis. Evavold et al. demonstrated GSDMD-dependent IL-1β release from living macrophages without pyroptotic cell death [160]. Whether an analogous sublytic state contributes to podocyte dysfunction by altering cell architecture, actin cytoskeletal dynamics, or slit-diaphragm stability remains unresolved and should be regarded as a hypothesis rather than an established podocyte-specific mechanism. Figure 3 therefore presents a proposed mechanistic framework rather than a universally established sequence from NLRP3 activation to podocyte pyroptosis.

#### 2.4.5. Role of cGAS–STING and NLRP3 Inflammasome Signaling in Kidney Diseases

Direct evidence for a podocyte-intrinsic cGAS–STING axis is currently strongest in diabetic kidney disease. In cultured podocytes and diabetic or obese mouse models, metabolic and lipotoxic stress induces mitochondrial injury and BAX-dependent leakage of mitochondrial DNA into the cytosol. Cytosolic mitochondrial DNA activates cGAS–STING signaling and is associated with NF-κB-dependent inflammatory gene expression, reduced slit-diaphragm protein abundance, and podocyte injury [132]. These findings identify mitochondrial stress as an upstream mechanism linking metabolic injury to intrinsic inflammatory signaling in podocytes.

Genetic studies further support a pathogenic role for podocyte STING signaling in diabetic kidney disease. Podocyte-specific *Sting* deletion in STZ-induced diabetic mice attenuates podocyte injury, renal dysfunction, proteinuria, and renal inflammation. In this model, podocyte *Sting* deletion also reduces NLRP3 inflammasome activation and pyroptosis, whereas NLRP3 overexpression offsets the protective effects of STING deficiency [163]. Thus, STING can function upstream of NLRP3-associated inflammatory injury in hyperglycemic podocytes, although the relative contribution of canonical inflammasome activity, pyroptosis, and inflammasome-independent signaling requires further clarification.

Podocyte-specific NLRP3 perturbation models likewise support a context-dependent role for NLRP3 in diabetic glomerular injury. Podocyte-specific expression of the gain-of-function *Nlrp3A350V* variant aggravates albuminuria, mesangial expansion, and GBM thickening in hyperglycemic mice. Conversely, podocyte-specific deletion of *Nlrp3* or *Casp1* protects against diabetic kidney disease. The more complete protection conferred by *Nlrp3* deletion than by *Casp1* deletion suggests that podocyte NLRP3 can mediate both caspase-1-dependent inflammasome functions and inflammasome-independent effects, including regulation of autophagy [155].

This podocyte-specific evidence should be interpreted alongside the broader contribution of NLRP3 in other renal compartments. Global *Nlrp3* deficiency attenuates renal inflammation, fibrosis, albuminuria, glomerular hypertrophy, mesangial expansion, and glomerulosclerosis in diabetic mice [164]. However, global genetic models cannot distinguish direct podocyte effects from altered inflammasome activity in infiltrating immune cells, tubular cells, endothelial cells, mesangial cells, and interstitial compartments. The genetic models summarized in Table 4 provide a framework for distinguishing podocyte-intrinsic cGAS–STING and inflammasome functions from systemic and multicompartment renal effects. In particular, comparison of global, podocyte-specific, and gain-of-function models indicates that the phenotypic consequences of cGAS–STING, NLRP3, caspase-1, and RIPK3 perturbation depend on both the disease context and the cellular compartment in which these pathways are modified.

The functional relevance of canonical NLRP3 inflammasome activity in podocytes remains controversial. In contrast to studies reporting pathogenic podocyte NLRP3 activity in diabetic kidney disease, single-cell transcriptomic analyses, experiments in renal progenitor-derived podocyte-like cells, and studies in diabetic mice with podocyte-specific expression of the NLRP3-A350V variant did not support canonical inflammasome activity in the podocyte systems examined [156]. These divergent results indicate that NLRP3 expression, canonical inflammasome competence, caspase-1 activation, cytokine processing, and pyroptotic death should be evaluated as distinct endpoints rather than treated as interchangeable readouts of podocyte injury.

Puromycin aminonucleoside nephropathy provides a widely used experimental model of oxidant-dependent podocyte injury. PAN induces ROS-dependent DNA damage in podocytes in vitro and in vivo [165]. In cultured podocytes, PAN also disrupts F-actin organization and induces cytoskeletal injury [166]. In rodent PAN nephropathy, podocyte damage is associated with proteinuria and foot-process abnormalities [167]. However, direct evidence that PAN-induced podocyte injury is mediated by cytosolic mitochondrial DNA sensing through cGAS–STING or by canonical NLRP3 inflammasome activation remains insufficient. PAN nephropathy should therefore be regarded as a relevant oxidative-stress model rather than as direct evidence for a podocyte-intrinsic cGAS–STING–NLRP3 axis.

Beyond diabetic kidney disease, functional cGAS–STING–NLRP3 interactions have been reported in APOL1-associated podocytopathy. APOL1 risk variants promote mitochondrial dysfunction, cytosolic mitochondrial DNA release, and activation of STING and NLRP3-associated inflammatory signaling in podocytes. Genetic and pharmacological pathway interventions attenuate inflammatory podocyte injury in this setting [149]. In membranous nephropathy, complement activation can promote pyroptosis-associated injury in podocytes, supporting an additional disease context in which inflammasome-related signaling and gasdermin-mediated membrane damage may contribute to filtration-barrier dysfunction [154].

Lupus nephritis illustrates the context dependence of cGAS–STING signaling. In TLR-dependent murine lupus models, global deletion of *Cgas* or *Sting* did not confer uniform protection and was associated with mildly exacerbated systemic autoimmune disease [145]. These findings do not establish a podocyte-specific role for cGAS–STING in lupus nephritis; rather, they caution that the consequences of systemic pathway inhibition may differ from those of cell-selective intervention in diabetic podocyte injury.

Together, current evidence identifies cGAS–STING and NLRP3 as context-dependent contributors to mitochondrial-stress responses, inflammation, and regulated cell-death susceptibility in podocytes. The most compelling evidence for a podocyte-intrinsic STING–NLRP3 axis is currently available in selected diabetic models. Future studies using inducible, cell-type-specific genetic systems and direct measures of inflammasome assembly, caspase-1 activity, gasdermin cleavage, cytokine maturation, and podocyte survival will be required to define the disease settings in which this pathway is a tractable therapeutic target.

**Table 4:** Genetic models defining cGAS–STING, inflammasome, and regulated cell-death functions in glomerular and podocyte injury.

Mediator	Genetic Strategy/Experimental Approach	Kidney Disease or Injury Model	Principal Findings	Reference
STING	Global deletion	HFD/STZ-induced diabetic kidney disease	STING deficiency attenuated renal fibrosis and reduced ID1-associated epithelial-to-mesenchymal transition. The protective phenotype was linked primarily to tubular epithelial responses; the direct contribution of podocytes and other renal cell types cannot be resolved in this global model	[168]
STING	Podocyte-specific deletion (Nphs2-Cre)	STZ-induced diabetes	Reduced podocyte damage, renal dysfunction, and inflammation, together with attenuation of NLRP3 inflammasome activation and pyroptosis	[163]
cGAS	Global deletion	MRL/*lpr* lupus model	Did not protect against lupus disease and was associated with a mildly exacerbated disease phenotype, demonstrating that global cGAS deficiency is not uniformly protective	[145]
RIPK3	Global deletion	Nephrotoxic nephritis	No significant improvement in urinary albumin-to-creatinine ratio, serum urea, glomerular thrombosis, macrophage infiltration, or glomerular apoptosis	[169]
RIPK3	Podocyte-specific deletion (*Podocin*-Cre)	Uninephrectomy plus HFD/STZ-induced diabetes	Reduced albuminuria, mesangial matrix expansion, podocyte foot-process injury, and podocyte loss are associated with reduced NF-κB p65-driven inflammation	[170]
NLRP3	Global deletion	STZ-induced diabetes	Reduced albuminuria and renal injury, with attenuation of glomerular hypertrophy, glomerulosclerosis, mesangial expansion, interstitial fibrosis, inflammation, and TGF-β1 expression	[164]
NLRP3	Podocyte-specific gain-of-function *Nlrp3A350V* expression	Hyperglycemic diabetic mice	Exacerbated albuminuria, mesangial expansion, and GBM thickening, supporting a pathogenic role for podocyte NLRP3 signaling in diabetic glomerular injury.	[155]
NLRP3	Podocyte-specific deletion (Nphs2-Cre)	STZ-induced diabetes	Reduced albuminuria and diabetic glomerular injury; protection exceeded that observed after podocyte-specific *Casp1* deletion, consistent with both caspase-1-dependent and inflammasome-independent NLRP3 functions.	[155]
NLRP3	Podocyte-specific expression of the gain-of-function *Nlrp3A350V* variant	Diabetic mice	Did not demonstrate appreciable canonical NLRP3 inflammasome activity in podocytes or stress-induced secretion of mature IL-1β and IL-18, indicating context-dependent inflammasome competence in kidney epithelial cells.	[156]
Caspase-1	Podocyte-specific deletion (*Nphs2-Cre*)	STZ-induced diabetes	Reduced diabetic glomerular injury and inflammasome-associated injury, but with less complete protection than podocyte-specific *Nlrp3* deletion.	[155]

Note: Podocyte-restricted genetic manipulation supports a contribution of the targeted pathway within podocytes but does not, by itself, establish an adult-specific function or independence from intercellular signaling. Global genetic manipulation does not establish podocyte specificity; developmental timing and inducibility should be considered when interpreting each model.

#### 2.4.6. ZBP1 as a Potential Amplifier of Podocyte Cell Death

Z-DNA-binding protein 1 (ZBP1), also known as DAI, is an interferon-inducible cytosolic sensor of nucleic acids in the Z conformation. ZBP1 recognizes Z-form nucleic acids, particularly Z-RNA, through two N-terminal Zα domains and signals through C-terminal RIP homotypic interaction motif (RHIM) domains that engage RIPK1 and RIPK3 [134,171]. In infection and inflammatory models, ZBP1 activation can initiate RIPK3-dependent MLKL phosphorylation, which, when it proceeds to MLKL oligomerization and membrane translocation, culminates in necroptosis. Depending on the stimulus and cellular context, ZBP1 can participate in the assembly of PANoptosome complexes containing RIPK1, RIPK3, caspase-8, and inflammasome components, thereby coordinating inflammatory cell-death responses [135,172,173].

Endogenous Z-RNAs represent an important potential source of ZBP1 activation. Under homeostatic conditions, ADAR1-mediated RNA editing restrains inappropriate sensing of endogenous double-stranded RNA and Z-form RNA species. Defects in ADAR1 function permit pathological ZBP1 activation and can cause RIPK3-MLKL-dependent necroptosis, inflammation, and PANoptosis [174,175]. ZBP1 is an interferon-inducible sensor; therefore, chronic interferon signaling may increase cellular susceptibility to ZBP1-dependent inflammatory cell death [134,176].

These features make ZBP1 a biologically plausible mediator of podocyte injury in disease settings characterized by interferon signaling, viral nucleic acid sensing, mitochondrial stress, or inflammatory activation. In particular, cGAS–STING activation may increase the expression of interferon-stimulated genes, including *ZBP1*, and could thereby lower the threshold for RIPK3-dependent death signaling. However, direct evidence that a cGAS–STING–ZBP1 axis drives podocyte death in vivo remains limited. ZBP1 expression, ligand engagement, assembly of a ZBP1-containing PANoptosome, and the relative contribution of apoptosis, necroptosis, and pyroptosis have not yet been systematically resolved in podocyte-specific genetic models.

RIPK3 provides a relevant downstream link between ZBP1 signaling and regulated cell death. In diabetic kidney disease, podocyte-specific *Ripk3* deletion reduces albuminuria, podocyte injury, mesangial matrix expansion, and NF-κB p65-driven inflammatory signaling [170]. Notably, high glucose did not increase MLKL phosphorylation in the experimental systems examined, and MLKL knockdown did not rescue podocyte injury, suggesting that RIPK3 can promote inflammatory damage independently of canonical MLKL-mediated necroptosis in diabetic podocytes [170]. These findings support a podocyte-intrinsic role for RIPK3 in diabetic injury but do not establish ZBP1 as its upstream activator in this setting.

Consistent with this evidence gap, the contribution of ZBP1 to podocyte injury remains insufficiently defined by podocyte-specific genetic evidence. Future studies should combine inducible podocyte-specific *Zbp1*, *Ripk3*, and *Mlkl* deletion with direct assessment of ZBP1 expression, Z-RNA accumulation, RIPK3/MLKL phosphorylation, caspase-8 activation, inflammasome assembly, gasdermin cleavage, and podocyte survival. Such experiments will be necessary to determine whether ZBP1 functions as a bona fide PANoptotic amplifier in podocytes or whether its major renal roles reside primarily in immune or tubular epithelial cells.

Across these signaling systems, redox regulation links metabolic stress, inflammatory activation, and podocyte fate. ROS are not uniformly harmful: their effects depend on the reactive species involved, their source and subcellular localization, and the balance between production and antioxidant defenses [177]. In cultured podocytes, AMPK-dependent protection against adriamycin-induced oxidative injury involves thioredoxin-mediated suppression of ASK1–p38 signaling [61]. In experimental diabetic podocytopathy, GSK3β hyperactivity impairs the NRF2 antioxidant response and promotes oxidative injury and senescence-like changes, whereas GSK3β inhibition or knockdown attenuates these effects [50]. Mitochondrial damage can additionally connect metabolic stress to inflammation through mitochondrial DNA leakage and cGAS–STING activation [132]. Evidence of redox imbalance or increased ROS does not, by itself, establish a causal role for a particular ROS source. Likewise, oxidative injury should be distinguished from ferroptosis, which requires iron-dependent lethal lipid peroxidation [178,179].

## 3. From Inflammatory Signaling to Podocyte Fate: Regulated Cell Death, Detachment, and Senescence

Activation of a death-associated pathway is not equivalent to execution of a defined death modality. Accordingly, inflammasome activation is distinguished from gasdermin-dependent pyroptotic death, RIPK3 signaling from MLKL-dependent necroptosis, and general oxidative injury from iron-dependent lethal lipid peroxidation. Simultaneous detection of apoptotic, pyroptotic, and necroptotic markers does not, by itself, establish functionally integrated PANoptosis [135]. In this review, autophagy, senescence, and detachment are considered separately from demonstrated cell death.

The inflammatory, metabolic, mitochondrial, and mechanical stress pathways described above do not operate in isolation. Podocytes integrate these inputs through interconnected networks that regulate cytoskeletal organization, mitochondrial homeostasis, survival, detachment, and senescence. Apoptosis, pyroptosis, necroptosis, and ferroptosis have been investigated as contributors to podocyte loss, although the strength of evidence varies among modalities and disease models. These processes intersect with autophagic dysfunction, PANoptosis-associated responses, and SASP programs.

**Figure 4:**
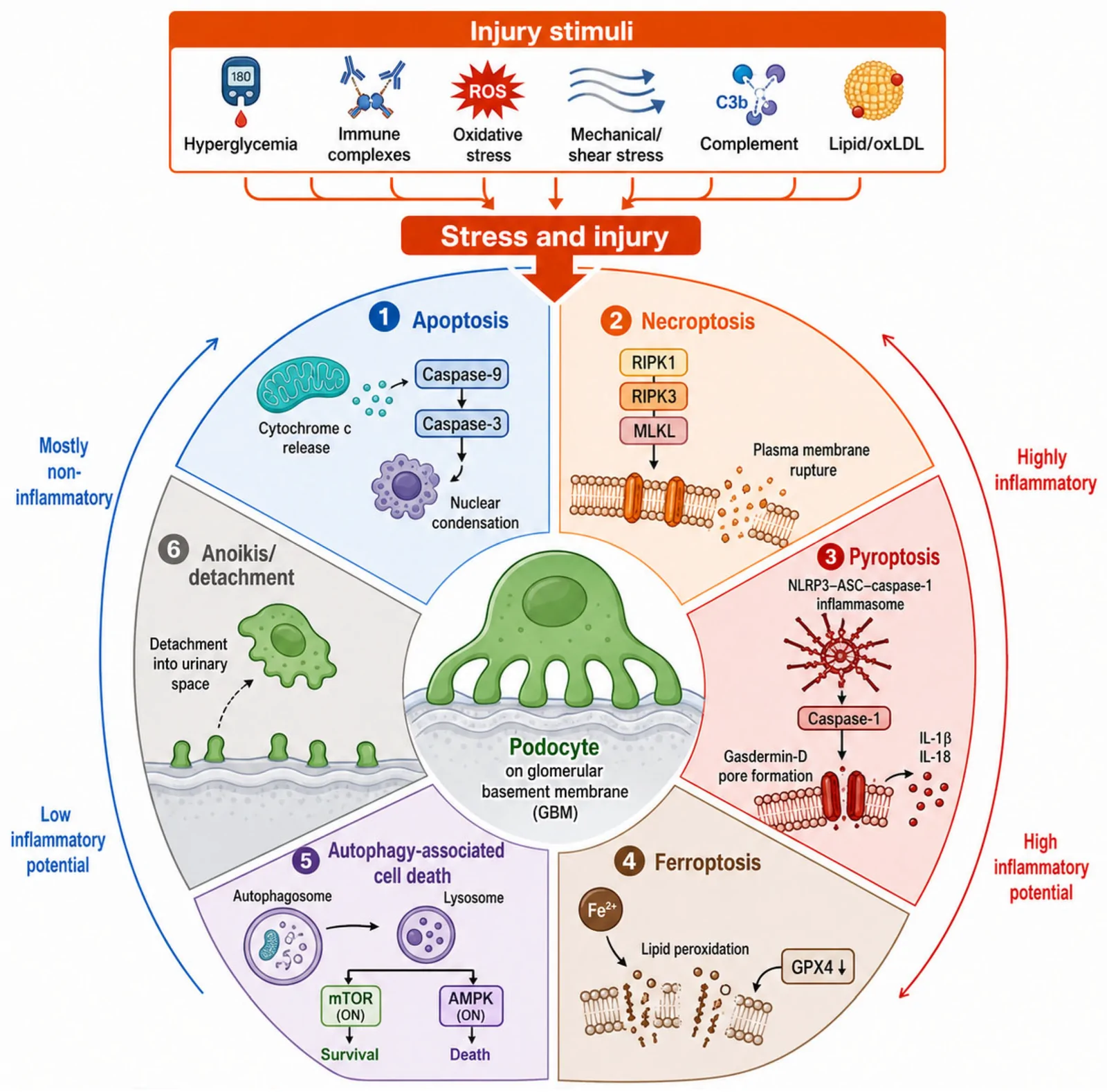
Podocyte fate under sustained inflammatory and metabolic stress: regulated cell death, detachment, and senescence. The schematic summarizes cell-death pathways and non-lethal cell states that may be engaged during podocyte injury; their involvement and degree of experimental validation vary among disease models. Intrinsic apoptosis involves mitochondrial outer membrane permeabilization and downstream caspase activation. Necroptosis is executed through RIPK3-dependent activation of MLKL, whereas the requirement for RIPK1 depends on the initiating stimulus. The canonical pyroptotic pathway depicted involves inflammasome-associated caspase-1 activation and GSDMD cleavage, although pathway activation or pore formation alone does not establish terminal lytic death. Ferroptosis is driven by iron-dependent accumulation of lethal lipid peroxides and can be favored by failure of glutathione- and GPX4-dependent antioxidant defenses. Autophagy generally supports podocyte homeostasis, while its dysregulation can modify susceptibility to injury without necessarily constituting a death mechanism. Podocyte detachment can occur before terminal cell death and may lead to podocyturia and depletion from the filtration barrier; anoikis refers specifically to death induced by loss of matrix attachment. Senescence-like changes include persistent DNA-damage responses, mitochondrial dysfunction, and inflammatory secretory reprogramming beyond the normal post-mitotic state. Concurrent activation of pyroptotic, apoptotic, and necroptotic components may indicate overlapping injury responses, but attribution to PANoptosis requires evidence of functional integration. Membrane rupture may permit damage-associated molecular pattern release, although its direct attribution to defined podocyte death modalities remains incompletely established. Metabolic, hemodynamic, immune, and mechanical stressors can influence these outcomes. Arrow notation: Solid black arrows indicate the direction of the depicted signaling steps, molecular events, or cellular outcomes; the dashed black arrow indicates podocyte detachment into the urinary space. Orange arrows indicate the convergence of injury stimuli on cellular stress and injury. The outer blue and red curved arrows indicate the broadly lower and higher inflammatory potential, respectively, of the depicted outcomes; they do not represent transitions between cell-death pathways or a quantitative scale. The downward arrow next to GPX4 indicates reduced GPX4-dependent antioxidant defense. Pathway arrows summarize reported or proposed relationships and do not imply that every step has been established in podocytes.

Podocyte fate reflects the interaction of stress intensity and duration with mitochondrial integrity and antioxidant capacity, rather than activation of a single signaling pathway. Experimental studies link impaired NRF2-dependent defenses to podocyte injury and senescence-like changes, whereas mitochondrial DNA leakage can engage cGAS–STING-dependent inflammatory injury. The availability and activation of death-execution machinery also matter: gasdermin cleavage must be distinguished from terminal lytic death [157,158,159,160], RIPK3 can promote injury independently of MLKL-mediated necroptosis, and ferroptosis requires iron-dependent lipid peroxidation to reach lethal levels. Matrix adhesion provides a separate determinant, as impaired adaptation to mechanical stress can promote podocyte detachment. These observations do not define a universal decision tree; different responses may coexist and their relative contributions require disease- and stage-specific evaluation. Figure 4 summarizes these potential outcomes and the stress signals that may influence their engagement.

### 3.1. Apoptosis (Caspase-9/3, Mitochondria)

Podocyte apoptosis has been demonstrated in experimental models of diabetic kidney disease and other proteinuric glomerular disorders, although its quantitative contribution to podocyte depletion in vivo remains difficult to establish [180]. Oxidative stress, endoplasmic-reticulum stress, and dysregulation of pro- and anti-apoptotic BCL-2 family proteins can promote mitochondrial outer membrane permeabilization, cytochrome c release, apoptosome assembly, and activation of caspase-9 and caspase-3. In diabetic experimental systems, oxidative mitochondrial injury is associated with cytochrome c release, Apaf-1-dependent apoptosome formation, caspase-9 activation, and podocyte apoptosis [180,181].

Podocyte apoptosis may be difficult to quantify in vivo because apoptotic cells can detach from the GBM and be lost into the urinary space before typical nuclear fragmentation is captured in tissue sections. Assessment should therefore combine podocyte identification with complementary morphological and biochemical evidence of apoptosis; reduced podocyte-marker abundance or cell number alone does not establish apoptotic death.

### 3.2. Pyroptosis (Gasdermin D, Caspase-1)

In selected diabetic and complement-associated experimental models, NLRP3–caspase-1–GSDMD signaling has been associated with podocyte injury, membrane permeabilization, proteinuria, and impairment of the glomerular filtration barrier [154,155]. Podocyte-specific *Nlrp3* and *Casp1* deletion attenuate diabetic glomerular injury, supporting a contributory role for NLRP3-associated signaling in this disease context [155]. However, canonical inflammasome competence, mature IL-1β/IL-18 secretion, and the extent of bona fide pyroptotic death in podocytes remain disputed and should be distinguished from inflammatory signaling in intrarenal immune cells [156]. Accordingly, increased expression of NLRP3, caspase-1, or GSDMD alone should not be interpreted as proof of podocyte pyroptosis. Robust attribution requires evidence of gasdermin cleavage and terminal lytic death in identified podocytes, supported by genetic or appropriately controlled pharmacological perturbation of the relevant execution machinery [157,158,159,160].

### 3.3. Necroptosis (RIPK3–MLKL Signaling)

Necroptosis is a regulated form of lytic cell death classically mediated by RIPK3-dependent phosphorylation and oligomerization of MLKL. ZBP1 can function upstream of RIPK3 in viral and interferon-rich inflammatory settings, although this mechanism has been established primarily outside the podocyte context [134,171].

In diabetic kidney disease, RIPK3 has a demonstrable podocyte-intrinsic pathogenic role. Podocyte-specific *Ripk3* deletion reduces albuminuria, mesangial matrix expansion, foot-process injury, podocyte loss, and NF-κB p65-driven inflammation [170]. However, high-glucose exposure did not increase MLKL phosphorylation in the systems examined, and MLKL knockdown did not rescue podocyte injury, indicating that RIPK3 can drive diabetic podocyte injury independently of canonical MLKL-mediated necroptosis [170]. Thus, RIPK3 signaling per se does not constitute necroptosis; in podocytes, its pathogenic role may be MLKL-independent. Direct evidence for ZBP1-dependent necroptosis in podocytes in vivo remains limited.

### 3.4. Ferroptosis (GPX4 and Lipid Peroxidation)

Ferroptosis is an iron-dependent form of regulated cell death characterized by the accumulation of lipid peroxides to lethal levels and failure of antioxidant defense systems centered on the system Xc^−^–glutathione–GPX4 axis [178,179]. In podocytes, emerging evidence from experimental FSGS and lupus nephritis supports an association between impaired GPX4/SLC7A11-related antioxidant defenses, glutathione depletion, iron accumulation, lipid peroxidation, and structural injury [182,183,184]. However, the disease-specific causal contribution of ferroptosis to podocyte loss remains under active investigation.

In ADR-associated FSGS models, reduced GPX4 expression in podocytes and pharmacological protection by ferroptosis inhibition have been reported, although the evidence base remains predominantly preclinical [182]. More recently, single-cell and integrated metabolic analyses in diabetic kidneys identified a podocyte-associated MAPK14–SLC7A11–GPX4 axis linked to ferroptosis-related injury [185].

Therapeutically, several preclinical interventions have been reported to attenuate renal and podocyte injury through GPX4-related antioxidant pathways. In experimental diabetic nephropathy, ginkgolide B inhibited GPX4 ubiquitination and restored GPX4 and FTH1 expression, whereas curcumol attenuated podocyte ferroptosis through the xCT/GPX4 axis [186,187]. In lupus nephritis, mesenchymal stromal cells reduced podocyte ferroptosis and improved podocyte structural markers through activation of the Nrf2/HO-1/GPX4 pathway [188]. These findings identify GPX4-related antioxidant defenses as experimentally tractable therapeutic checkpoints. However, these findings remain preclinical and do not establish ferroptosis as a universally dominant mechanism of podocyte loss across proteinuric kidney diseases. Changes in ROS, GPX4 abundance, or lipid peroxidation alone are insufficient for attribution; stronger evidence combines demonstrable cell death with lipid-peroxidation measurements and rescue by mechanistically appropriate ferroptosis inhibitors or genetic interventions [178].

### 3.5. PANoptosis and the PANoptosome

PANoptosis is an integrated inflammatory cell-death program in which pyroptotic, apoptotic, and necroptotic mechanisms are coordinated through multiprotein PANoptosome complexes. Depending on the initiating stimulus and cellular context, PANoptosomes can incorporate sensors and signaling molecules such as ZBP1, RIPK1, RIPK3, FADD, caspase-8, inflammasome components, and caspase-1, thereby enabling concurrent or interdependent activation of pyroptosis, apoptosis, and necroptosis [135,172].

Lv et al. reported TRAIL–DR5-dependent PANoptosis-associated injury in experimental diabetic kidney disease [189]. In cultured podocytes, TRAIL or DR5 silencing reduced the reported death-associated responses, whereas TRAIL overexpression or receptor activation enhanced them. Podocyte-specific deletion of the TRAIL or DR5 genes also attenuated podocyte and glomerular injury in diabetic mice [189]. These functional interventions support a pathogenic role for the TRAIL–DR5 axis beyond marker coexpression alone, while the molecular organization and functional integration of the proposed podocyte PANoptosome require further clarification.

The precise composition of the podocyte PANoptosome and the contribution of individual upstream sensors remain incompletely resolved. In particular, direct evidence that cGAS–STING activation or ZBP1-dependent Z-RNA sensing nucleates a bona fide PANoptosome in podocytes in vivo is currently limited. cGAS–STING, NLRP3, RIPK3, and death-receptor signaling may nevertheless create a cellular environment permissive for overlapping pyroptotic, apoptotic, and necroptotic pathways in diabetic and inflammatory podocytopathies. Future studies should determine whether these pathways converge within a common multiprotein complex or instead represent parallel, partially overlapping injury programs.

Across these death-associated pathways, extracellular damage-associated molecular pattern (DAMP) signaling should be distinguished from intracellular danger sensing. Gao et al. detected increased HMGB1 in the culture supernatant of LPS-exposed mouse podocytes, but did not establish whether its release required pyroptosis, necroptosis, ferroptosis, or another death mechanism. The study also showed injury-associated responses in HK-2 cells exposed to exogenously added HMGB1, rather than demonstrating transfer of injury through podocyte-conditioned medium. These findings support extracellular HMGB1 accumulation during podocyte stress, but do not resolve the release mechanism or establish death-mode-specific inflammatory propagation. Establishing such a mechanism would require pathway-specific perturbation coupled to extracellular DAMP measurements; demonstrating downstream inflammatory effects would additionally require recipient-cell assays and DAMP-specific blockade [190].

### 3.6. Podocyte Detachment, Anoikis-like Death, and Podocyturia

Podocyte detachment from the GBM is a major route to podocyte depletion and podocyturia, but it does not necessarily coincide with cell death [191,192,193,194,195,196,197]. Attachment depends on integrin-containing adhesion complexes mechanically coupled to the actin cytoskeleton, including complexes containing talin and α-parvin. These adhesions function together with cytoskeletal regulators and slit-diaphragm-associated signaling, including α-actinin-4 and nephrin–Nck pathways, to maintain podocyte structural integrity [191,192,193,194].

Mechanical stress, altered matrix signaling, cytoskeletal disorganization, and disruption of integrin-associated adhesions can reduce podocyte mechanical resilience and promote detachment. Disease-causing ACTN4 variants provide a genetic illustration of this process: mutant α-actinin-4 impairs adaptation of podocytes to repeated mechanical stretch and increases susceptibility to detachment [193]. Similarly, genetic disruption of parvin-dependent integrin adhesions impairs podocyte mechano-adaptation and promotes detachment, with β-parvin providing a compensatory mechanism in the absence of α-parvin [194].

Detachment-induced cell death may involve anoikis-like mechanisms, but podocyte detachment should not be treated as a molecularly uniform form of cell death. Viable podocytes can be recovered from urine, indicating that detachment and terminal cell death are not invariably synchronous events [195,196]. The term “podoptosis” has been used in the podocyte literature, but it should be applied cautiously because it has also been used to describe p53-dependent podocyte cell death rather than detachment alone [197].

Complement activation, oxidative stress, and inflammatory mediators can destabilize podocyte architecture by perturbing the actin cytoskeleton, slit-diaphragm components, and plasma-membrane integrity. In membranous nephropathy, complement-associated injury can perturb podocyte mitochondria and cytoskeletal organization; C3a- and C5a-induced pyroptosis-associated responses have also been reported in cultured podocytes [28,154]. Thus, podocyte detachment is best regarded as a final common structural endpoint of multiple upstream inflammatory, metabolic, mechanical, and cytoskeletal injuries rather than as evidence for one specific regulated cell-death pathway.

### 3.7. Podocyte Senescence and Senescence-Associated Inflammatory Signaling

Beyond acute cell-death modalities, podocytes can acquire senescence-like changes characterized by persistent DNA-damage responses, mitochondrial dysfunction, altered chromatin regulation, and inflammatory secretory reprogramming. Because mature podocytes are already post-mitotic, lack of proliferation alone cannot distinguish senescence from their normal differentiated state. In aging experimental systems, increased p16 expression and senescence-associated β-galactosidase activity accompany inflammatory transcriptional programs involving NLRP3, IL-6, TNF, and interferon responses [198].

NLRP3 signaling contributes to inflammaging in podocytes. In middle-aged mice, systemic MCC950 treatment or global *Nlrp3* deletion reduced podocyte senescence-associated markers and was accompanied by higher podocyte density, improved foot-process morphology, and preservation of podocyte differentiation markers [198]. These findings support a role for NLRP3-associated inflammatory signaling in podocyte aging, although they do not establish that canonical inflammasome-mediated IL-1β/IL-18 secretion is the principal mechanism in podocytes.

Sirtuin- and epitranscriptomic pathways further modulate podocyte stress resilience. Podocyte-specific SIRT1 overexpression and pharmacological SIRT1 activation attenuated diabetes-induced podocyte loss, oxidative stress, albuminuria, and glomerular injury in diabetic mice [199]. Conversely, podocyte-specific *Mettl14* deletion mitigated ADR-associated glomerulopathy and podocyte injury, in part through preservation of *Sirt1* expression, enhanced autophagy, and reduced apoptosis and inflammatory signaling [100].

These data identify senescence-like reprogramming as a distinct but interconnected outcome of chronic metabolic, oxidative, and inflammatory stress in podocytes. However, the composition, secretion, and paracrine activity of a podocyte-specific SASP remain incompletely resolved across disease contexts. Future studies should distinguish senescence-like reprogramming from the normal post-mitotic state, dedifferentiation, autophagic dysfunction, and pre-terminal injury using complementary molecular, structural, and functional criteria.

## 4. Disease Context and Therapeutic Implications

**Figure 5:**
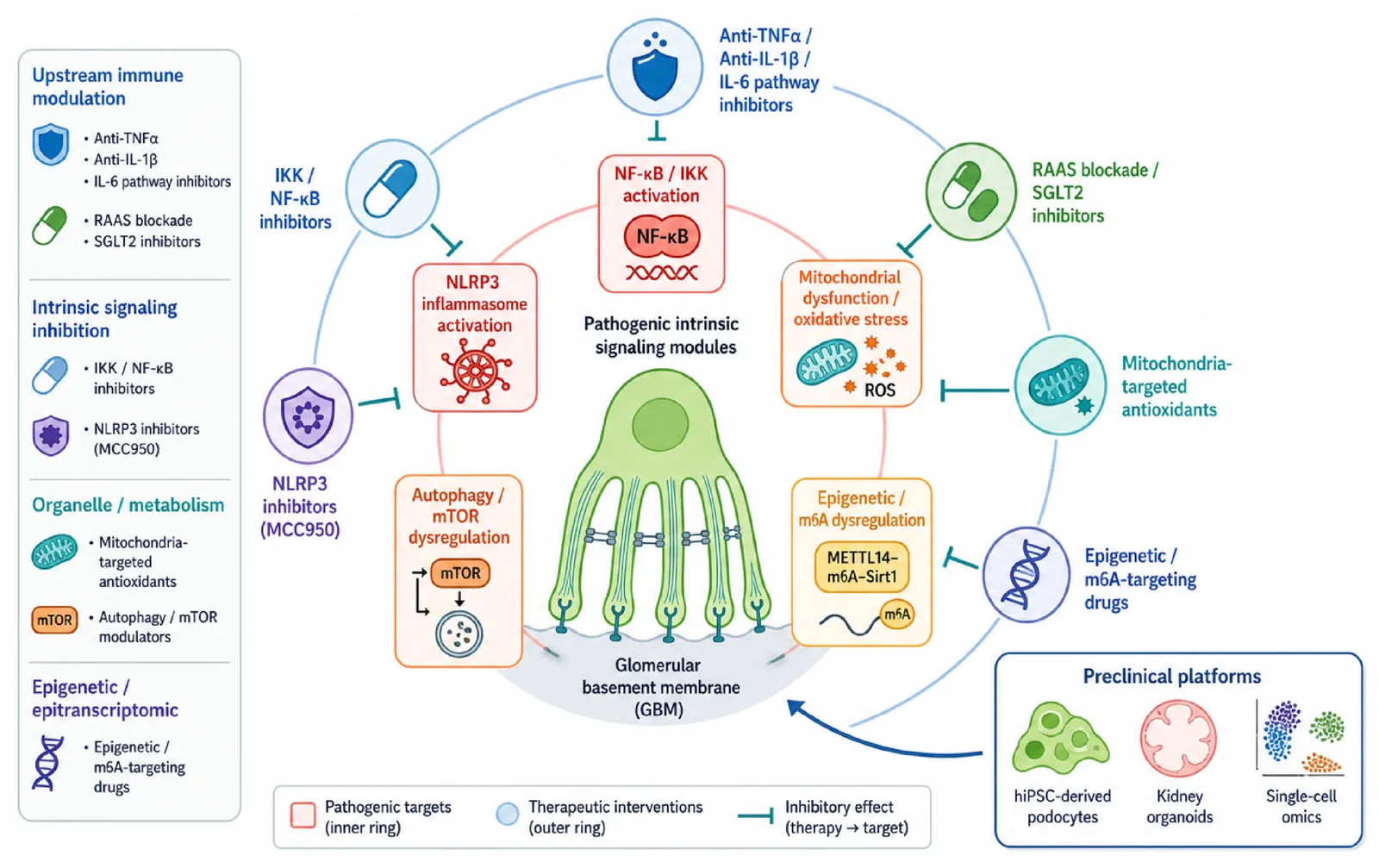
Therapeutic entry points along the podocyte-intrinsic inflammatory signaling network. Podocyte injury can be targeted at three complementary levels. Upstream interventions reduce hemodynamic, metabolic, cytokine, complement, and lipid-associated stress that activates podocyte receptors and cytosolic danger-sensing pathways. Established kidney-protective therapies, including renin–angiotensin system blockade, SGLT2 inhibitors, and, in appropriate settings, non-steroidal mineralocorticoid receptor antagonists, act predominantly through systemic and intraglomerular mechanisms and may indirectly reduce upstream drivers of podocyte injury. Intracellular inflammatory and cytoskeletal nodes, including NF-κB, JAK–STAT, cGAS–STING, NLRP3, Rho–ROCK, mitochondrial stress, autophagy, and epigenetic regulators, represent potential targets for experimental pathway-directed intervention. Downstream strategies may aim to limit cytoskeletal dysfunction, inflammatory cell death, detachment, and senescence-associated secretory phenotypes SASP. Most intracellular pathway inhibitors remain preclinical, and their therapeutic benefit will depend on disease stage, target engagement, cell-type specificity, pharmacokinetics, and preservation of essential homeostatic and host-defense functions. Human podocyte models, kidney organoids, glomerulus-on-chip systems, and single-cell or spatial profiling can support mechanistic validation, target identification, and biological patient stratification for future podocyte-targeted therapies.

As summarized in Figure 5, podocyte-intrinsic inflammatory signaling presents therapeutic opportunities at three complementary levels: reduction in upstream hemodynamic, metabolic, cytokine, and complement stress; context-dependent modulation of intracellular inflammatory and cytoskeletal pathways; and limitation of downstream cell-death and senescence-associated programs. The therapeutic goal is not complete suppression of NF-κB, p38 MAPK, JAK–STAT, STING, NLRP3, or Rho-family GTPase signaling, because these pathways also contribute to stress adaptation, cytoskeletal homeostasis, and host defense. Instead, the available evidence favors partial, disease-stage-specific attenuation of maladaptive pathway activation, ideally combined with cell-selective delivery strategies.

Inhibitors of the inflammatory signaling pathways discussed in this review are summarized in Table S1, including their targets, disease models, reported efficacy, limitations, and clinical status. Renal improvement following systemic treatment should not, by itself, be interpreted as evidence of direct podocyte-specific target engagement.

Established kidney-protective therapy should remain the therapeutic foundation. Renin–angiotensin system blockade, SGLT2 inhibitors, and, in appropriate patients with type 2 diabetes and chronic kidney disease, the non-steroidal mineralocorticoid receptor antagonist finerenone reduce albuminuria and improve kidney outcomes [200,201,202]. Their clinical benefit cannot be assigned exclusively to podocytes. Nevertheless, by lowering intraglomerular pressure and improving the metabolic, oxidative, and inflammatory milieu of the kidney, these agents are expected to reduce major upstream drivers of podocyte injury. They should therefore be viewed as systemic kidney-protective therapies that may indirectly constrain podocyte-intrinsic inflammatory signaling, rather than as selective podocyte pathway inhibitors. In experimental diabetic kidney disease, canagliflozin has also been reported to attenuate TXNIP/NLRP3-associated inflammatory signaling and pyroptotic injury in podocytes; this observation requires distinction from the established clinical outcome evidence for the SGLT2 inhibitor class [203].

At the cytokine and JAK–STAT level, anti-cytokine biologics and JAK inhibitors may reduce the inflammatory burden imposed on podocytes in autoimmune or cytokine-rich disease states, but these therapies are not podocyte-specific and can compromise protective immune responses. Baricitinib provides proof of pharmacological and translational plausibility rather than established clinical efficacy in diabetic kidney disease: in a Phase II randomized trial in type 2 diabetes with diabetic kidney disease, baricitinib 4 mg daily reduced the morning urinary albumin-to-creatinine ratio by 41% relative to placebo at Week 24 [204]. Whether baricitinib modifies long-term kidney-function decline or kidney-failure risk in diabetic kidney disease remains unknown. Experimental data support a local IL-6–gp130–JAK2–STAT3 circuit in high-glucose-exposed podocytes: high glucose increased IL-6 production, JAK2 activation, nuclear STAT3 phosphorylation, and cellular hypertrophy, whereas IL-6 neutralization attenuated these responses in vitro [78]. Experimental studies further support a pathogenic role for excessive STAT3 activity in diabetic glomerulopathy and inflammatory kidney injury [79]. However, the therapeutic value of direct STAT3 inhibition in diabetic kidney disease remains uncertain because the available evidence is predominantly preclinical, and current small-molecule inhibitors, including Stattic and S3I-201, have unresolved limitations in selectivity, tissue distribution, and long-term safety; no STAT3-directed therapy has been established for glomerular disease [205].

Inhibition of intracellular inflammatory sensors is mechanistically attractive but remains preclinical. In diabetic and obesity-associated experimental models, mitochondrial DNA leakage activates a podocyte cGAS–STING/TBK1/p65 inflammatory axis. Genetic STING suppression and pharmacological inhibition reduced podocyte injury and improved renal phenotypes; C-176 reduced albuminuria and podocyte injury in diet-induced obesity mice [132]. In complementary work, podocyte-specific *Sting* deletion ameliorated inflammation, renal dysfunction, and podocyte injury in STZ-diabetic mice, whereas H-151 improved renal inflammation and function in *db/db* mice [163]. However, no cGAS or STING inhibitor has established clinical efficacy in glomerular disease, and species-specific STING pharmacology must be considered, particularly for C-176.

NLRP3 inhibition is supported by substantial mechanistic and preclinical evidence. In *db/db* mice, MCC950 improved renal function and attenuated albuminuria, mesangial expansion, podocyte loss, and glomerular lipid accumulation, whereas NLRP3 deficiency produced concordant protection in STZ-induced diabetes [206]. In high-glucose-exposed podocytes, NLRP3 silencing or MCC950 reduced apoptosis, cytoskeletal remodeling, mitochondrial ROS generation, NF-κB p65 activation, and lipid accumulation, consistent with an IL-1β/ROS/NF-κB p65-associated mechanism of inflammasome-driven podocyte injury [206]. MCC950 has also been associated with improvements in GBM thickening, foot-process fusion, and podocyte-marker expression in diabetic mice [207]. However, results are not uniform. In a delayed-intervention model of established STZ-induced diabetic kidney disease, MCC950 increased renal inflammation and structural injury, including mesangial expansion and glomerulosclerosis [208]. Treatment timing, disease stage, model selection, pharmacokinetics, and the relative contribution of immune-cell versus intrinsic glomerular inflammasome activity are therefore likely to determine the response to NLRP3 inhibition. MCC950 and related agents should be classified as experimental NLRP3-targeted candidates, not yet clinically validated podocyte-directed therapies. The divergent results obtained with MCC950 emphasize that pharmacological NLRP3 inhibition cannot be interpreted solely as a podocyte-directed intervention, because systemic exposure may modify inflammasome-related responses in infiltrating immune, tubular, endothelial, and mesangial compartments.

The p38 MAPK and Rho–ROCK axes provide complementary therapeutic entry points because they connect inflammatory transcriptional programs to cytoskeletal destabilization, foot-process effacement, and detachment. In human glomerulonephritis, p38 MAPK activation and cellular localization correlate with histological and functional indices of renal injury [209]. Although p38 inhibition has shown benefit in selected experimental renal and glomerular injury models, its effects are model- and cell-context-dependent, and systemic p38 inhibitors have encountered translational constraints, including safety limitations. Accordingly, they should not yet be considered an established class of glomerular therapeutics. By contrast, pharmacological Rho-kinase inhibition with fasudil reduced renal ROCK activity, albuminuria, mesangial matrix expansion, collagen IV accumulation, and glomerular basement membrane GBM thickening in *db/db* mice without significantly altering blood glucose levels [210]. Genetic data provide a more selective rationale for ROCK2: podocyte-specific ROCK2 deletion protected against albuminuria, renal hypertrophy, mesangial expansion, podocyte loss, foot-process widening, and glomerular basement membrane GBM thickening across STZ-induced, *db/db,* and high-fat-diet models of diabetes [123]. Mechanistically, ROCK2 deficiency restored PPARα-associated fatty-acid oxidation and improved podocyte energy homeostasis, identifying ROCK2 as a potential metabolic as well as cytoskeletal regulator of diabetic podocytopathy [123]. Fasudil is clinically available in Japan for the management of cerebral vasospasm after aneurysmal subarachnoid hemorrhage, but it is not an approved treatment for diabetic kidney disease or glomerular disease; it should therefore be regarded as a repurposing candidate rather than a validated glomerular therapy [211].

Epigenetic and epitranscriptomic regulators, including the METTL14–m6A–SIRT1 axis, sirtuins, histone-modifying enzymes, and inflammation-associated non-coding RNAs, offer potential means of reprogramming persistent stress and senescence-like phenotypes in podocytes [100,199,212]. These targets remain hypothesis-generating because their functions are strongly cell- and context-dependent, and indiscriminate interference with chromatin or RNA-modifying enzymes may disrupt podocyte homeostasis as well as maladaptive stress responses. Their prioritization requires podocyte-restricted genetic validation, pharmacodynamic evidence of renal target engagement, and targeted-delivery approaches.

Overall, the most immediately translatable approach is to combine guideline-based kidney-protective therapy with carefully selected pathway-directed interventions in biologically stratified disease settings. Progress will require evidence of target engagement in urinary podocytes or kidney biopsy material, separation of podocyte-autonomous from systemic immune effects, and validation in human podocyte, organoid, and glomerulus-on-chip systems. The central challenge is therefore not simply the identification of additional inhibitors but the matching of pathway activity, disease stage, and delivery modality to the inflammatory state of the podocyte.

## 5. Conclusions and Unresolved Questions

The evidence reviewed here supports a revised view of podocytes as active, context-dependent participants in glomerular inflammation rather than passive structural targets. Podocytes possess cell-autonomous stress-sensing and inflammatory signaling mechanisms that can influence filtration-barrier integrity, cytoskeletal homeostasis, and cellular fate. NF-κB and p38 MAPK function as complementary transcriptional and stress-kinase modules, whereas JAK–STAT relays cytokine inputs, cGAS–STING and NLRP3 respond to intracellular danger signals, and Rho-family GTPases translate inflammatory and metabolic cues into cytoskeletal remodeling. When sustained or dysregulated, this interconnected network can shift from adaptive stress responses toward maladaptive inflammation, senescence-like reprogramming, regulated cell death, detachment, and progressive podocyte depletion. Despite substantial progress, several fundamental questions remain.

Five priorities emerge from the available evidence. Causality should be tested through functional perturbation and supported where feasible by rescue experiments, rather than inferred from pathway-marker changes alone. Cell specificity requires lineage-restricted approaches and direct assessment of target engagement in the relevant cellular compartment. Temporal regulation should be addressed using adult-inducible and time-resolved designs that distinguish adaptive responses from sustained injury. Human translation requires validation in appropriately characterized human-derived systems and clinical tissue, with attention to cellular maturation and disease context. Therapeutic selectivity requires separation of podocyte effects from systemic immune, vascular, and tubular actions, together with evaluation of the consequences of disrupting homeostatic signaling.

Future research could leverage hiPSC-derived podocytes, kidney organoids, glomerulus-on-chip systems, and CRISPR/Cas9-based isogenic models to dissect podocyte-intrinsic pathways in human genetic and disease contexts. Such platforms can enable controlled interrogation of candidate regulators of inflammation, mitochondrial stress, regulated cell death, and senescence-like reprogramming. Single-cell multi-omics and spatial transcriptomics should further refine the molecular definition of podocyte injury states across diabetic kidney disease, lupus nephritis, and IgA nephropathy, APOL1-associated disease, FSGS, and hypertensive nephropathy. These approaches, combined with longitudinal analysis of urinary podocytes and blood or urine biomarkers, could help identify disease-stage-specific signatures of pathway activity and inform biologically stratified clinical studies.

Ultimately, the goal is to move beyond broadly immunosuppressive or non-selective pathway inhibition toward more precise, podocyte-informed therapeutic strategies. Such approaches should preserve glomerular filtration-barrier integrity by rebalancing intrinsic inflammatory signaling, stabilizing cytoskeletal and mitochondrial homeostasis, and limiting maladaptive cell death, detachment, and senescence-like programs.

## Figures and Tables

**Table 2:** Genetic models defining JAK–STAT pathway functions in podocytes and glomerular disease.

Mediator	Genetic Manipulation	Experimental Model or Context	Principal Phenotype	Reference
JAK2	Germline deletion	Developmental phenotype	Embryonic lethality at approximately E12.5 caused by severe anemia and failure of definitive erythropoiesis; adult renal phenotypes cannot be assessed in conventional knockout mice.	[97,98]
JAK2	Podocyte-specific overexpression	Diabetic 129S6 mice with Akita diabetes and angiotensin II infusion	Increased albuminuria, mesangial expansion, glomerulosclerosis, fibronectin accumulation, GBM thickening, and podocyte loss; systemic JAK1/2 inhibition partially reverses the phenotype	[12]
STAT1	Germline deletion	MRL/lpr lupus nephritis	Reduced proteinuria and glomerulonephritis severity but aggravated Th17-associated tubulointerstitial inflammation, accompanied by a shift toward STAT3/STAT4 signaling; this model does not establish a podocyte-autonomous STAT1 function.	[99]
STAT3	Podocyte-specific conditional deletion (*Nphs2*-Cre)	Nephrotoxic serum-induced crescentic glomerulonephritis	Reduced proteinuria, renal functional decline, crescent formation, glomerular proliferation, loss of podocyte markers, and glomerular expression of inflammatory STAT3 target genes.	[79]
STAT3	Germline deletion	Developmental phenotype	Early embryonic lethality at E6.5–E7.5; adult renal phenotypes cannot be assessed.	[96]
STAT3	Podocyte-specific conditional deletion	Tg26 HIV-1 transgenic model of HIV-associated nephropathy	Reduced albuminuria, renal functional impairment, glomerular collapse and sclerosis, podocyte proliferation and dedifferentiation, inflammatory STAT3 target-gene expression, and secondary tubulointerstitial injury.	[94]
STAT5	Podocyte-specific conditional deletion	ADR-induced FSGS-like nephropathy	Increased albuminuria, podocyte flattening, foot-process effacement, and nephrin loss indicate a protective STAT5-dependent podocyte response	[85]
IL-23R	Podocyte-specific conditional deletion	MRL/lpr lupus nephritis	Reduced clinical and histological manifestations of lupus nephritis and podocyte injury; IL-23 exposure induced podocyte cytoskeletal remodeling and pSTAT3 accumulation.	[92]

Note: Podocyte-restricted genetic manipulation supports a contribution of the targeted pathway within podocytes but does not, by itself, establish an adult-specific function or independence from intercellular signaling. Global genetic manipulation does not establish podocyte specificity; developmental timing and inducibility should be considered when interpreting each model.

## Data Availability

No new data were created or analyzed in this study. Data sharing is not applicable to this article.

## Supplementary Materials

The following supporting information can be downloaded at: https://www.mdpi.com/article/10.3390/ijms27219923/s1, References [213,214,215,216,217,218,219,220,221,222,223,224,225,226,227,228,229,230,231,232,233,234,235,236,237,238,239,240,241,242] are cited in the supplementary materials.

## Abbreviations

The following abbreviations are used in this manuscript:

ADR	Adriamycin
AGE	Advanced glycation end product
AKI	Acute kidney injury
ANGPTL4	Angiopoietin-like protein 4
APOL1	Apolipoprotein L1
ASC	Apoptosis-associated speck-like protein containing a caspase recruitment domain
AT1R	Angiotensin II type 1 receptor
cGAMP	Cyclic guanosine monophosphate–adenosine monophosphate
cGAS	Cyclic GMP–AMP synthase
C5b-9	Complement membrane attack complex
CARD	Caspase recruitment domain
CCL2	C-C motif chemokine ligand 2
CD2AP	CD2-associated protein
COX-2	Cyclooxygenase-2
DAMP	Damage-associated molecular pattern
FSGS	Focal segmental glomerulosclerosis
GAP	GTPase-activating protein
GBM	Glomerular basement membrane
GEF	Guanine nucleotide exchange factor
GN	Glomerulonephritis
GSDMD	Gasdermin D
GSK3β	Glycogen synthase kinase 3 beta
GPX4	Glutathione peroxidase 4
HFD	high-fat diet
HIV	human immunodeficiency virus
IFN	Interferon
IRF3	Interferon regulatory factor 3
JAK	Janus kinase
LRR	Leucine-rich repeat
MAPK	Mitogen-activated protein kinase
METTL14	Methyltransferase-like 14
MLKL	Mixed lineage kinase domain-like protein
MRL/lpr	Murphy Roths Large/lymphoproliferation mouse strain
MyD88	Myeloid differentiation primary response 88
NACHT	NAIP, CIITA, HET-E and TP1 domain
NEK7	NIMA-related kinase 7
NEMO	NF-κB essential modulator
NET	Neutrophil extracellular trap
NF-κB	Nuclear factor kappa B
NIK	NF-κB-inducing kinase
NLRP3	NOD-like receptor family pyrin domain-containing 3
NPHS1	Nephrin
NPHS2	Podocin
PAMP	Pathogen-associated molecular pattern
PANoptosis	Pyroptosis, apoptosis, and necroptosis
p65	NF-κB RelA subunit
PI3K	Phosphoinositide 3-kinase
pMLKL	Phosphorylated mixed lineage kinase domain-like protein
PRR	Pattern-recognition receptor
PYD	Pyrin domain
RAC1	Ras-related C3 botulinum toxin substrate 1
RAGE	Receptor for advanced glycation end products
RAAS	Renin–angiotensin–aldosterone system
RCD	Regulated cell death
REDD1	Regulated in development and DNA damage response 1
RHIM	RIP homotypic interaction motif
ROCK	Rho-associated coiled-coil-containing protein kinase
ROS	Reactive oxygen species
SASP	Senescence-associated secretory phenotype
scRNA-seq	Single-cell RNA sequencing
SGLT2	Sodium-glucose cotransporter 2
SIRT1	Sirtuin 1
SIRT6	Sirtuin 6
SLC7A11	Solute carrier family 7 member 11
SOCS3	Suppressor of cytokine signaling 3
STING	Stimulator of interferon genes
STZ	Streptozotocin
TBK1	TANK-binding kinase 1
TGF-β	Transforming growth factor beta
TLR	Toll-like receptor
TNF	Tumor necrosis factor
TRAF	TNF receptor-associated factor
ZBP1	Z-DNA-binding protein 1
