# Supplementary material for: Intrinsic Inflammatory Signaling in Life and Death of Podocytes
Source: Int J Mol Sci. 2026 Nov 5;27(21):9923. doi: 10.3390/ijms27219923 (PMC13764566; doi:10.3390/ijms27219923)
Supplement: Supplementary file 1 [file ijms-27-09923-s001.zip › ijms-4613661-supplementary.pdf]

Supplementary Table S1. Selected pharmacological modulators of inflammatory signaling in glomerular disease and related kidney injury models

Inhibitor / Drug	Target	Disease model and evidence type	Efficacy	Limitations and podocyte specificity	Clinical status
**NF-κB signaling pathway**					
Pyrrolidine dithiocarbamate	Non-specific NF-κB/redox signaling modulation	Renal ischemia–reperfusion (Wistar rats); in vivo pharmacological study, systemic treatment.	reduced biochemical and histological evidence of renal dysfunction and injury, the expression of inducible nitric oxide synthase protein and serum levels of nitric oxide [1]	Non-specific NF-κB/redox modulation; potential toxicity. Whole-kidney outcomes do not establish a direct podocyte-specific effect.	preclinical
Sappanone A	NF-κB/IκBα	STZ-induced diabetes after uninephrectomy (C57BL/6J mice); in vivo pharmacology plus high-glucose-treated glomerular mesangial cells in vitro.	attenuated the pathological changes, reduced serum creatinine and blood urea nitrogen, alleviated TGF-β1, Collagen-IV production, decreased inflammatory cytokines [2]	Limited preclinical evidence. Cell experiments used mesangial cells, not podocytes; direct podocyte-specific target engagement was not established.	preclinical
BAY 11-7082	NF-κB pathway inhibition; additional NLRP3-inhibitory activity [4]	STZ-induced diabetes (Sprague-Dawley rats); in vivo pharmacological study, systemic treatment.	improved renal histological architecture and reduced inflammatory cytokine levels [3]	Non-specific pathway effects and potential concentration-dependent cytotoxicity. Systemic studies [3,4] do not establish direct podocyte-specific target engagement.	preclinical
		Lupus nephritis (MRL/lpr mice); in vivo pharmacological study, systemic treatment.	attenuated renal damage, suppressed NLRP3 inflammasome/IL-1β pathway in the kidneys, inhibited the production of TNF, reduced renal CCL2 expression and macrophage infiltration [4]		
TDZD-8	GSK3β	Lupus nephritis (MRL/lpr mice); systemic in vivo pharmacology, with supporting macrophage experiments in vitro.	inhibited the activation of GSK-3*β*, caspase 1, with a decrease in interleukin-1*β* [5]	Limited preclinical evidence; supporting cellular data concern macrophages, not podocytes. Direct podocyte-specific target engagement was not established.	preclinical
SRT1720	SIRT1 agonist	ADR-induced glomerulosclerosis (BALB/c mice); in vivo pharmacological study reported as a conference abstract [6].	improved renal function, reduced proteinuria [6]	Conference abstract, not a full-length study report. Nephrin staining supports a glomerular readout, but direct podocyte-specific target engagement was not established.	Preclinical

**p38 MAPK signaling pathway**					
SB202190	p38α/β MAPK	STZ-induced acute hyperglycemia (C57BL/6 mice); systemic in vivo pharmacology plus mechanistic studies in cultured murine podocytes and HEK293T cells.	preserves nephrin surface expression and significantly attenuates albuminuria [7]	Acute experimental model; pharmacological selectivity and systemic effects limit attribution. Podocyte/nephrin mechanistic evidence is available, but in vivo drug action was not restricted to podocytes.	preclinical
SB203580	p38α/β MAPK	Lupus nephritis (MRL/lpr mice); in vivo pharmacological study, systemic treatment.	improved renal function by decreasing the levels of proteinuria, serum BUN, ameliorating the pathologic changes of kidney, reducing Ig and C(3) depositions [8]	Systemic immunomodulation and limited preclinical evidence; direct podocyte-specific target engagement was not established.	preclinical
VX-745 (Neflamapimod)	p38α MAPK	No renal efficacy study cited; clinical studies concern neurological indications [35].	not tested	No renal efficacy study is cited in this table; applicability to glomerular disease and podocyte-specific target engagement remain unestablished.	Investigational; clinical development in neurological indications [35]. Renal efficacy and podocyte-specific target engagement have not been established.
Losmapimod	p38α/β MAPK	Idiopathic FSGS (adults); single-arm, open-label, multicenter phase II trial (17 treated participants) [9].	No participant achieved the prespecified primary endpoint in the phase II FSGS trial; the study was terminated after a prespecified interim analysis [9]	Small, uncontrolled study with a heterogeneous FSGS population; no serious adverse events were reported. Direct podocyte-specific target engagement was not established.	Phase II study in FSGS terminated after a prespecified interim analysis with a negative primary efficacy result [9].
**JAK-STAT signaling pathway**					
Baricitinib	JAK1/2	Diabetic kidney disease in adults with type 2 diabetes; randomized, double-blind, placebo-controlled, dose-ranging phase II trial (129 participants) [34].	Baricitinib 4 mg daily reduced morning urinary albumin-to-creatinine ratio by 41% versus placebo at week 24 and decreased urinary CXCL10 and CCL2 and circulating inflammatory biomarkers [10,11,34].	Anemia was more frequent with 4 mg daily than placebo. Albuminuria and inflammatory biomarkers are surrogate outcomes; long-term	Phase II randomized trial in diabetic kidney disease; renal efficacy remains investigational, with no established long-term kidney-

				renal benefit and direct podocyte-specific target engagement were not established [34].	outcome benefit [34].
Tofacitinib	JAK1/3	Pristane-induced lupus nephritis (BALB/c mice); in vivo pharmacological study, systemic treatment.	reduced proteinuria, anti-dsDNA, IL-6, IFN-γ, reduction in glomerulonephritis, delayed the nephritic physiological progression [12]	Systemic immunomodulation and infection risk; the cited murine study does not establish direct podocyte-specific target engagement.	Clinical use in other indications; evidence in the renal models listed is preclinical
Ruxolitinib	JAK1/2	STZ-induced diabetes (Wistar rats); in vivo pharmacological study, systemic treatment.	reduction in albuminuria, renal injury, inflammation and fibrosis [13]	Myelosuppression and infection risk with systemic JAK inhibition. Renal data concern whole-kidney, fibroblast, and tubular-cell effects; direct podocyte-specific target engagement was not established.	Clinical use in other indications; evidence in the renal models listed is preclinical.
		Unilateral ureteral obstruction (C57BL/6 mice); systemic in vivo pharmacology plus TGF-β1-treated renal fibroblasts and tubular epithelial cells in vitro.	reduction in tubular injury, blocking renal fibroblast activation, inflammation and reduced renal tubular epithelial cell apoptosis [14]		
Filgotinib	JAK1	Lupus membranous nephropathy (patients); small randomized, double-blind phase II trial comparing filgotinib with lanraplenib [36]; summarized in review [15].	Median reduction in 24-hour urine protein of 50.7% at week 16 among four filgotinib-treated participants completing that visit; the very small sample permits only exploratory conclusions [15,36].	Very small human trial (5 assigned filgotinib; 4 evaluated at week 16) and conference-abstract evidence for the mouse model. Systemic JAK inhibition; neither study establishes direct podocyte-specific target engagement [16,36].	Phase II exploratory study in lupus membranous nephropathy; mouse renal evidence is preclinical. Renal efficacy is not established [16,36].
		Lupus nephritis (NZB/W F1 mice); systemic in vivo pharmacology, immune-cell phenotyping, and whole-blood target-coverage assay; conference abstract [16].	decrease in proteinuria, reduction in BUN levels, renal inflammation, improved glomerular morphology, and increased survival [16]		
AG-490	JAK2	Type 1 diabetes (NOD mice); in vivo diabetes-prevention/reversal study with supporting immune-cell experiments; not a renal efficacy study.	Prevented diabetes development and induced remission in newly diabetic NOD mice; renal efficacy was not assessed [17].	Renal and podocyte outcomes were not evaluated in the cited study; diabetes prevention/reversal must not be interpreted as treatment of glomerular disease.	Preclinical diabetes evidence only; no renal efficacy assessment in the cited study.

S3I-201	STAT3	STZ-induced diabetes (C57BL/6 mice); systemic in vivo pharmacology plus renal STAT3 knockdown and high-glucose-treated tubular epithelial cells in vitro.	reduction in albuminuria, renal injury, expression of TGF-β1, ACE/AT1, VEGF in diabetic kidneys [18]	Limited selectivity and off-target effects. Mechanistic evidence is predominantly tubular; neither cited renal model establishes direct podocyte-specific target engagement.	preclinical
		Lupus nephritis (MRL/lpr mice); in vivo pharmacological study focused on tubulointerstitial injury [19]; additional context from review [20].	reduction in albuminuria, decreased interstitial fibrosis [19,20]		
Stattic	STAT3	Lupus nephritis (MRL/lpr mice); systemic in vivo pharmacology plus T-cell proliferation and migration assays in vitro.	reduction in albuminuria, glomerular IgG deposition, injury, lymphadenopathy, lower levels of anti-dsDNA antibody [21]	Systemic immunosuppression and reduced T-cell populations; supporting cell experiments used T cells, not podocytes. Direct podocyte-specific target engagement was not established.	preclinical
**Rho-family GTPase signaling**					
Fasudil	ROCK1/ROCK2	STZ-induced diabetes (C57BL/6 mice); in vivo pharmacological study with macrophage-polarization readouts.	decreased urinary protein and serum creatinine, but not blood glucose; increased the number of M2-type macrophages and anti-inflammatory cytokines, which attenuated renal injury [22]	Systemic ROCK inhibition may affect vascular tone and immune cells. The listed studies do not establish direct podocyte-specific target engagement.	Preclinical evidence in the renal models listed.
		Type 2 diabetes (db/db mice); in vivo pharmacological study, systemic treatment.	reduced albuminuria, glomerular collagen IV accumulation, and urinary collagen IV excretion [23]		
Y-27632	ROCK1/ROCK2	Angiotensin II-induced renal damage (Wistar rats); in vivo pharmacological study, systemic treatment.	ameliorates renal damage, decreases the upregulation of these proinflammatory and profibrotic mediators [24]	Systemic ROCK inhibition and off-target effects limit mechanistic attribution. The listed renal studies do not establish direct podocyte-specific target engagement.	preclinical
		Renal ischemia–reperfusion (Sprague-Dawley rats); in vivo pharmacological study, systemic treatment.	administration before ischemia attenuated the ischemia/reperfusion-induced renal dysfunction and histological damage, such as tubular necrosis [25]		

NSC23766	Rac1	Renal ischemia–reperfusion (C57BL/6 mice); systemic in vivo pharmacology plus IL-4-treated mouse monocytes in vitro.	alleviated renal damage and dysfunction, reduced profibrotic molecules levels [26]	Limited preclinical evidence; supporting cellular data concern monocytes/macrophages. Direct podocyte-specific target engagement was not established.	preclinical
**Cytosolic nucleic-acid sensing and inflammasome signaling pathway**					
C-176	STING	Type 2 diabetes (db/db mice); systemic in vivo pharmacology plus lipotoxicity-stressed cultured podocytes and STING knockdown in vitro [27].	reduced albuminuria and podocyte injury in the reported mouse models [27]	Murine STING inhibitor; human translation is unestablished. Cultured-podocyte pharmacology and STING knockdown support an intrinsic effect in [27], but systemic in vivo treatment is not podocyte-specific; [28] concerns macrophages.	preclinical
		Diet-induced obesity (mice); systemic in vivo pharmacology, supported by the cultured-podocyte experiments in the same study [27].	reduced albuminuria, glomerular hypertrophy, improved glucose tolerance, insulin tolerance and podocyte injury [27]		
		LPS-induced acute kidney injury (C57BL/6J mice); systemic in vivo pharmacology plus LPS/IFN-γ-induced macrophage polarization in vitro [28].	alleviated acute kidney injury and improved renal function, with reduced macrophage infiltration, M1 polarization, and inflammatory responses [28]		
MCC950	NLRP3	Type 2 diabetes (db/db mice); systemic in vivo pharmacology plus high-glucose-treated rat glomerular mesangial cells in vitro [29].	reduced serum creatinine, attenuated mesangial expansion with increased matrix and tubular dilatation, alleviated thickened glomerular basement membrane and foot process fusion, decreased interstitial fibrosis [29]	Mesangial-cell assays and in vivo podocyte markers/ultrastructure do not establish direct podocyte-specific drug engagement. Renal evidence in this table is preclinical.	preclinical
CY-09	NLRP3	Type 2 diabetes (db/db mice); in vivo pharmacology plus supporting high-glucose cell experiments in vitro [30].	reduced inflammation, oxidative stress, fibrosis, apoptosis and level of caspase-1, IL-18, IL-1β [30]	Limited preclinical evidence; the cited study does not establish podocyte-specific target engagement or clinical renal efficacy.	preclinical
Tranilast	NLRP3-inhibitory activity reported in the literature;	Diabetic kidney disease (Sprague-Dawley rats); in	reduced renal interstitial fibrosis through inhibition of mast cell infiltration mediated through the SCF/c-kit signaling pathway	The cited rat experiment concerns mast cells and	Clinical use for allergic conditions in

	mast-cell/SCF–c-kit-associated effects in the cited renal study [31,32]	vivo pharmacology focused on mast-cell infiltration and interstitial fibrosis [31]; mechanistic context from review [32].	[31,32]	interstitial fibrosis, not direct podocyte-specific NLRP3 inhibition; the NLRP3 rationale is discussed in review [32].	some jurisdictions; evidence in the renal model listed is preclinical.
Brilliant Blue G	P2X7	Unilateral ureteral obstruction (Wistar rats); in vivo pharmacological study of early renal inflammation and fibrosis.	showed lower expression of procollagens and IL-1β, less immunoreactivity of HSP-47, TGF-β, macrophages, myofibroblasts, and tubular apoptosis [33]	Early obstructive-injury model with tubular/interstitial readouts; direct podocyte-specific target engagement was not established.	preclinical

Note: The table includes studies of glomerular disease and selected related renal injury models. Reported efficacy refers to the outcomes measured in the cited studies and does not necessarily establish direct podocyte-specific target engagement. Clinical development or approval in another indication should not be interpreted as established efficacy or approval for the renal condition listed.

References

1. Chatterjee, P.K.; D'Emmanuele di Villa Bianca, R.; Sivarajah, A.; McDonald, M.C.; Cuzzocrea, S.; Thiemermann, C. Pyrrolidine dithiocarbamate reduces renal dysfunction and injury caused by ischemia/reperfusion of the rat kidney. *Eur. J. Pharmacol.* **2003**, *482*, 271–280. https://doi.org/10.1016/j.ejphar.2003.09.071
2. Wang, Z.; Chen, Z.; Wang, X.; Hu, Y.; Kong, J.; Lai, J.; Li, T.; Hu, B.; Zhang, Y.; Zheng, X.; et al. Sappanone A prevents diabetic kidney disease by inhibiting kidney inflammation and fibrosis via the NF-κB signaling pathway. *Front. Pharmacol.* **2022**, *13*, 953004. https://doi.org/10.3389/fphar.2022.953004
3. Kolati, S.R.; Kasala, E.R.; Bodduluru, L.N.; Mahareddy, J.R.; Uppulapu, S.K.; Gogoi, R.; Barua, C.C.; Lahkar, M. BAY 11-7082 ameliorates diabetic nephropathy by attenuating hyperglycemia-mediated oxidative stress and renal inflammation via NF-κB pathway. *Environ. Toxicol. Pharmacol.* **2015**, *39*, 690–699. https://doi.org/10.1016/j.etap.2015.01.019
4. Zhao, J.; Zhang, H.; Huang, Y.; Wang, H.; Wang, S.; Zhao, C.; Liang, Y.; Yang, N. BAY 11-7082 attenuates murine lupus nephritis via inhibiting NLRP3 inflammasome and NF-κB activation. *Int. Immunopharmacol.* **2013**, *17*, 116–122. https://doi.org/10.1016/j.intimp.2013.05.027
5. Zhao, J.; Wang, H.; Huang, Y.; Zhang, H.; Wang, S.; Gaskin, F.; Yang, N.; Fu, S.M. Lupus nephritis: Glycogen synthase kinase 3β promotion of renal damage through activation of the NLRP3 inflammasome in lupus-prone mice. *Arthritis Rheumatol.* **2015**, *67*, 1036–1044. https://doi.org/10.1002/art.38993
6. Lopes Gonçalves, G.; Oliveira-Souza, M. SRT1720 Administration Attenuates Albuminuria and Renal Fibrosis on Adriamycin-Induced Glomerulosclerosis. *FASEB J.* **2021**, *35*. https://doi.org/10.1096/fasebj.2021.35.S1.01762 [Conference abstract].
7. Woznowski, M.P.; Potthoff, S.A.; Königshausen, E.; et al. Inhibition of p38 MAPK decreases hyperglycemia-induced nephrin endocytosis and attenuates albuminuria. *J. Mol. Med.* **2022**, *100*, 781–795. https://doi.org/10.1007/s00109-022-02184-5
8. Jin, N.; Wang, Q.; Zhang, X.; Jiang, D.; Cheng, H.; Zhu, K. The selective p38 mitogen-activated protein kinase inhibitor, SB203580, improves renal disease in MRL/lpr mouse model of systemic lupus. *Int. Immunopharmacol.* **2011**, *11*, 1319–1326. https://doi.org/10.1016/j.intimp.2011.04.015
9. Gipson, D.S.; Hladunewich, M.A.; Lafayette, R.A.; Sedor, J.R.; Rovin, B.H.; Barbour, S.J.; McMahon, A.; Jennette, J.C.; Nachman, P.H.; Willette, R.N.; et al. Assessing the Impact of Losmapimod on Proteinuria in Idiopathic Focal Segmental Glomerulosclerosis. *Kidney Int. Rep.* **2020**, *5*, 1228–1239. https://doi.org/10.1016/j.ekir.2020.05.024
10. Brosius, F.C.; Tuttle, K.R.; Kretzler, M. JAK inhibition in the treatment of diabetic kidney disease. *Diabetologia* **2016**, *59*, 1624–1627. https://doi.org/10.1007/s00125-016-4021-5

11. Liu, Y.; Wang, W.; Zhang, J.; Gao, S.; Xu, T.; Yin, Y. JAK/STAT signaling in diabetic kidney disease. *Front. Cell Dev. Biol.* **2023**, *11*, 1233259. https://doi.org/10.3389/fcell.2023.1233259
12. Lin, J.; Zhang, Y.; Wang, M.; Zhang, Y.; Li, P.; Cao, Y.; Yang, X. Therapeutic Effects of Tofacitinib on Pristane-Induced Murine Lupus. *Arch. Rheumatol.* **2022**, *37*, 195–204. https://doi.org/10.46497/ArchRheumatol.2022.8252
13. El-Kady, M.M.; Naggar, R.A.; Guimei, M.; Talaat, I.M.; Shaker, O.G.; Saber-Ayad, M. Early Renoprotective Effect of Ruxolitinib in a Rat Model of Diabetic Nephropathy. *Pharmaceuticals* **2021**, *14*, 608. https://doi.org/10.3390/ph14070608
14. Bai, Y.; Wang, W.; Yin, P.; Gao, J.; Na, L.; Sun, Y.; Wang, Z.; Zhang, Z.; Zhao, C. Ruxolitinib Alleviates Renal Interstitial Fibrosis in UUO Mice. *Int. J. Biol. Sci.* **2020**, *16*, 194–203. https://doi.org/10.7150/ijbs.39024
15. Torun, E.S. Efficacy and Safety of Janus Kinase Inhibitors in Lupus Nephritis Patients: Clinical Trials and Case Reports. *Med. Res. Arch.* **2024**, *12*. https://doi.org/10.18103/mra.v12i12.6221
16. Pohlmeyer, C.; Cui, Z.; Han, P.; et al. AB0484 Monotherapy with filgotinib, a JAK1-selective inhibitor, reduces disease severity and alters immune cell subsets in the NZB/W F1 murine model of lupus. *Ann. Rheum. Dis.* **2018**, *77*, 1403. https://doi.org/10.1136/annrheumdis-2018-eular.3367 [Conference abstract].
17. Davoodi-Semiromi, A.; Wasserfall, C.H.; Xia, C.Q.; Cooper-DeHoff, R.M.; Wabitsch, M.; et al. The Tyrphostin Agent AG490 Prevents and Reverses Type 1 Diabetes in NOD Mice. *PLoS ONE* **2012**, *7*, e36079. https://doi.org/10.1371/journal.pone.0036079
18. Zheng, C.; Huang, L.; Luo, W.; et al. Inhibition of STAT3 in tubular epithelial cells prevents kidney fibrosis and nephropathy in STZ-induced diabetic mice. *Cell Death Dis.* **2019**, *10*, 848. https://doi.org/10.1038/s41419-019-2085-0
19. Du, Y.; Zhang, W.; Liu, S.; Feng, X.; Gao, F.; Liu, Q. S3I-201 ameliorates tubulointerstitial lesion of the kidneys in MRL/lpr mice. *Biochem. Biophys. Res. Commun.* **2018**, *503*, 177–180. https://doi.org/10.1016/j.bbrc.2018.05.207
20. Pace, J.; Paladugu, P.; Das, B.; He, J.C.; Mallipattu, S.K. Targeting STAT3 signaling in kidney disease. *Am. J. Physiol. Renal Physiol.* **2019**, *316*, F1151–F1161. https://doi.org/10.1152/ajprenal.00034.2019
21. Edwards, L.J.; Mizui, M.; Kyttaris, V. Signal transducer and activator of transcription (STAT) 3 inhibition delays the onset of lupus nephritis in MRL/lpr mice. *Clin. Immunol.* **2015**, *158*, 221–230. https://doi.org/10.1016/j.clim.2015.04.004
22. Xie, F.; Lei, J.; Ran, M.; Li, Y.; Deng, L.; Feng, J.; Zhong, Y.; Li, J. Attenuation of Diabetic Nephropathy in Diabetic Mice by Fasudil through Regulation of Macrophage Polarization. *J. Diabetes Res.* **2020**, *2020*, 4126913. https://doi.org/10.1155/2020/4126913
23. Kolavennu, V.; Zeng, L.; Peng, H.; Wang, Y.; Danesh, F.R. Targeting of RhoA/ROCK Signaling Ameliorates Progression of Diabetic Nephropathy Independent of Glucose Control. *Diabetes* **2008**, *57*, 714–723. https://doi.org/10.2337/db07-1241
24. Rupérez, M.; Sánchez-López, E.; Blanco-Colio, L.M.; Esteban, V.; Rodríguez-Vita, J.; Plaza, J.J.; Egido, J.; Ruiz-Ortega, M. The Rho-kinase pathway regulates angiotensin II-induced renal damage. *Kidney Int.* **2005**, *68*, S39–S45. https://doi.org/10.1111/j.1523-1755.2005.09908.x
25. Teraishi, K.; Kurata, H.; Nakajima, A.; Takaoka, M.; Matsumura, Y. Preventive effect of Y-27632, a selective Rho-kinase inhibitor, on ischemia/reperfusion-induced acute renal failure in rats. *Eur. J. Pharmacol.* **2004**, *505*, 205–211. https://doi.org/10.1016/j.ejphar.2004.10.040
26. Liang, H.; Huang, J.; Huang, Q.; Xie, Y.C.; Liu, H.Z.; Wang, H.B. Pharmacological inhibition of Rac1 exerts a protective role in ischemia/reperfusion-induced renal fibrosis. *Biochem. Biophys. Res. Commun.* **2018**, *503*, 2517–2523. https://doi.org/10.1016/j.bbrc.2018.07.009
27. Zang, N.; Cui, C.; Guo, X.; Song, J.; Hu, H.; Yang, M.; Xu, M.; Wang, L.; Hou, X.; He, Q.; et al. cGAS-STING activation contributes to podocyte injury in diabetic kidney disease. *iScience* **2022**, *25*, 105145 https://doi.org/10.1016/j.isci.2022.105145
28. Leng, X.; Li, Q.; Chen, W.; Feng, H.; Li, L.; Yu, L.; Huang, P.; Ma, P.; Xie, F. C-176 inhibits macrophage polarization towards M1-subtype and ameliorates LPS induced acute kidney injury. *Eur. J. Pharmacol.* **2024**, *984*, 177028. https://doi.org/10.1016/j.ejphar.2024.177028

29. Zhang, C.; Zhu, X.; Li, L.; Ma, T.; Shi, M.; Yang, Y.; et al. A small molecule inhibitor MCC950 ameliorates kidney injury in diabetic nephropathy by inhibiting NLRP3 inflammasome activation. *Diabetes Metab. Syndr. Obes.* **2019**, *12*, 1297–1309. https://doi.org/10.2147/DMSO.S199802
30. Yang, M.; Zhao, L. The Selective NLRP3-Inflammasome Inhibitor CY-09 Ameliorates Kidney Injury in Diabetic Nephropathy by Inhibiting NLRP3-Inflammasome Activation. *Curr. Med. Chem.* **2023**, *30*, 3261–3270. https://doi.org/10.2174/0929867329666220922104654
31. Yin, D.D.; Luo, J.H.; Zhao, Z.Y.; Liao, Y.J.; Li, Y. Tranilast prevents renal interstitial fibrosis by blocking mast cell infiltration in a rat model of diabetic kidney disease. *Mol. Med. Rep.* **2018**, *17*, 7356–7364. https://doi.org/10.3892/mmr.2018.8776
32. Luo, Y.; Long, M.; Wu, X.; Zeng, L. Targeting the NLRP3 inflammasome in kidney disease: Molecular mechanisms, pathogenic roles, and emerging small-molecule therapeutics. *Front. Immunol.* **2025**, *16*, 1703560. https://doi.org/10.3389/fimmu.2025.1703560
33. Pereira, J.M.S.; Barreira, A.L.; Gomes, C.R.; et al. Brilliant blue G, a P2X7 receptor antagonist, attenuates early phase of renal inflammation, interstitial fibrosis and is associated with renal cell proliferation in ureteral obstruction in rats. *BMC Nephrol.* **2020**, *21*, 206. https://doi.org/10.1186/s12882-020-01861-2
34. Tuttle, K.R.; Brosius, F.C., III; Adler, S.G.; Kretzler, M.; Mehta, R.L.; Tumlin, J.A.; Tanaka, Y.; Haneda, M.; Liu, J.; Silk, M.E.; et al. JAK1/JAK2 inhibition by baricitinib in diabetic kidney disease: Results from a Phase 2 randomized controlled clinical trial. Nephrol. Dial. Transplant. 2018, 33, 1950–1959. https://doi.org/10.1093/ndt/gfx377
35. CervoMed. Neflamapimod Clinical Results. Available online: https://www.cervomed.com/clinical-results/ (accessed on 29 October 2026).
36. Baker, M.; Chaichian, Y.; Genovese, M.; Derebail, V.; Rao, P.; Chatham, W.; Bubb, M.; Lim, S.; Hajian, H.; Gurtovaya, O.; et al. Phase II, randomised, double-blind, multicentre study evaluating the safety and efficacy of filgotinib and lanraplenib in patients with lupus membranous nephropathy. RMD Open 2020, 6, e001490. https://doi.org/10.1136/rmdopen-2020-001490
